# The genome of okra (*Abelmoschus esculentus*) provides insights into its genome evolution and high nutrient content

**DOI:** 10.1093/hr/uhad120

**Published:** 2023-06-02

**Authors:** Ruyu Wang, Wei Li, Qiang He, Hongyu Zhang, Meijia Wang, Xinyuan Zheng, Ze Liu, Yu Wang, Cailian Du, Huilong Du, Longsheng Xing

**Affiliations:** College of Life Sciences, Institute of Life Sciences and Green Development, Hebei University, Baoding 071000, China; College of Life Sciences, Institute of Life Sciences and Green Development, Hebei University, Baoding 071000, China; Hebei Basic Science Center for Biotic Interaction, Institute of Life Sciences and Green Development, Hebei University, Baoding 071000, China; College of Life Sciences, Institute of Life Sciences and Green Development, Hebei University, Baoding 071000, China; Hebei Basic Science Center for Biotic Interaction, Institute of Life Sciences and Green Development, Hebei University, Baoding 071000, China; College of Life Sciences, Institute of Life Sciences and Green Development, Hebei University, Baoding 071000, China; College of Life Sciences, Institute of Life Sciences and Green Development, Hebei University, Baoding 071000, China; College of Life Sciences, Institute of Life Sciences and Green Development, Hebei University, Baoding 071000, China; College of Life Sciences, Institute of Life Sciences and Green Development, Hebei University, Baoding 071000, China; College of Life Sciences, Institute of Life Sciences and Green Development, Hebei University, Baoding 071000, China; College of Life Sciences, Institute of Life Sciences and Green Development, Hebei University, Baoding 071000, China; College of Life Sciences, Institute of Life Sciences and Green Development, Hebei University, Baoding 071000, China; Hebei Basic Science Center for Biotic Interaction, Institute of Life Sciences and Green Development, Hebei University, Baoding 071000, China; College of Life Sciences, Institute of Life Sciences and Green Development, Hebei University, Baoding 071000, China; Hebei Basic Science Center for Biotic Interaction, Institute of Life Sciences and Green Development, Hebei University, Baoding 071000, China

## Abstract

Okra (*Abelmoschus esculentus*) is an important vegetable crop with high nutritional value. However, the mechanism underlying its high nutrient content remains poorly understood. Here, we present a chromosome-scale genome of okra with a size of 1.19 Gb. Comparative genomics analysis revealed the phylogenetic status of *A. esculentus*, as well as whole-genome duplication (WGD) events that have occurred widely across the Malvaceae species. We found that okra has experienced three additional WGDs compared with the diploid cotton *Gossypium raimondii*, resulting in a large chromosome number (2n = 130). After three WGDs, okra has undergone extensive genomic deletions and retained substantial numbers of genes related to secondary metabolite biosynthesis and environmental adaptation, resulting in significant differences between okra and *G. raimondii* in the gene families related to cellulose synthesis. Combining transcriptomic and metabolomic analysis, we revealed the relationship between gene expression and metabolite content change across different okra developmental stages. Furthermore, the sinapic acid/S-lignin biosynthesis-related gene families have experienced remarkable expansion in okra, and the expression of key enzymes involved in the sinapic acid/S-lignin biosynthesis pathway vary greatly across developmental periods, which partially explains the differences in metabolite content across the different stages. Our study gains insights into the comprehensive evolutionary history of Malvaceae species and the genetic basis that underlies the nutrient content changes in okra, which will facilitate the functional study and genetic improvement of okra varieties.

## Introduction

Okra (*Abelmoschus esculentus* L.), also known as lady’s fingers and belonging to the family Malvaceae, is extensively cultivated in tropical and subtropical areas around the world and represents a horticultural crop greatly favored by people [1]. Okra is also commonly used for curing chronic dysentery, gonorrhea, urinary incontinence, strangury, and diarrhea [2]. Recently, pharmacological studies have revealed the widespread antioxidant, anti-inflammatory, immunomodulatory, lipid-lowering, anticancer, antimicrobial, and antidiabetic activities of okra [3, 4]. Dietary supplementation with okra polysaccharides reduces body weight, blood glucose, and total serum cholesterol level in C57BL/6 mice with a high-fat diet [5]. The medicinal potential of okra is attributed mainly to its rich bioactive substances, such as phenolic acids, alkaloids, and flavonoids [6].

Phenolic acids are a class of crucial secondary metabolites that are commonly distributed in higher plants, and they show potency for the treatment of various diseases such as diabetes, cardiovascular and neurodegenerative diseases, and cancer [7]. Sinapic acid, one of the most important phenolic acids, has anticancer, anti-oxidant, cardioprotective, neuroprotective, and anti-diabetic activities, which may be significantly related to the curative effects of okra [8]. Previously, it has been reported that metabolites and nutrient contents show dynamic changes during different growth and developmental stages of plants [9–11]. To date, the pattern of change of the sinapic acid content during okra development, and the genetic mechanisms underlying the changes in nutrient content in okra pods at different developmental stages, remain unclear.

Whole-genome duplications (WGDs) are an essential driving force for speciation and environmental adaptation in many lineages [12]. In addition to okra, the Malvaceae family also harbors many other important species. The genomes of multiple Malvaceae species have been published, mainly from two genera, *Gossypium* and *Hibiscus*, including *Gossypium raimondii* [13], *Gossypium darwinii* [14], *Gossypium herbaceum* [15], *Gossypium hirsutum* [16], *Hibiscus syriacus* [17], *Hibiscus cannabinus* [18], *Hibiscus mutabilis* [19], and *Hibiscus hamabo* [20]. The chromosome numbers in Malvaceae are highly variable [21], which might reflect the complex evolutionary history of this family. However, a systematic study of the process of evolution of Malvaceae species is still lacking. Therefore, there is an urgency to assemble a high-quality okra genome and perform a systematic study of Malvaceae species to explore speciation and evolution across the Malvaceae species. As one of the most representative Malvaceae species, cotton is recognized as the most significant economic crop for the production of natural fiber around the world. There are significant differences between cotton and okra, including plant morphology, metabolite content, and polysaccharide types. However, the genetic mechanisms that underlie these trait differences remain unclear.

In this study, we obtained 65 okra chromosomes by combining the Illumina, PacBio HiFi, and high-throughput chromosome conformation capture (Hi-C) sequencing technologies. The phylogenetic relationship of Malvaceae species was analyzed, and the association between the expanded gene families in okra and environmental adaptation and bioactive substance synthesis was investigated. Additionally, a systematic comparative analysis of the ancient WGD events was performed to reveal the evolutionary process and the complexity of chromosome numbers across the Malvaceae species. Based on transcriptome and metabolome data across different tissues and developmental stages, gene co-expression network analysis was performed to elucidate the network modules involved in the synthesis of bioactive substances and the key regulatory genes in the biosynthesis pathway. Overall, this study provides important genomic resources for evolutionary analysis of the Malvaceae family and okra functional genomics research.

## Results

### Chromosome-scale genome assembly and annotation of okra

To obtain the okra genome, 58.66 Gb HiFi long reads, 55.18 Gb Hi-C paired reads, and 94.59 Gb Illumina sequencing reads were obtained (Tables S1 and S2, see online supplementary material). The initial genome assembly was 1.19 Gb and consisted of 128 contigs with a contig N50 size of 16.98 Mb (Table 1; Table S2, see online supplementary material). The assembled genome size was approaching the estimated genome size based on flow cytometry (1.22 Gb) and k-mer analysis (1.23 Gb) (Fig. S1 and Table S3, see online supplementary material). These contigs were further corrected and anchored onto 65 pseudo-chromosomes (Fig. S2, see online supplementary material) encompassing 99.96% of the assembled sequences (Table S4, see online supplementary material), consistent with the results of fluorescence *in situ* hybridization (FISH) (Fig. S3, see online supplementary material). Of these, 36 chromosomes consisted of a single contig, while most of the remaining chromosomes harbored 1–2 gaps (Table S5, see online supplementary material). The DNA and RNA short reads were aligned with our assembled genome with an average mapping rate of 95.73% and 97.10%, respectively (Table S6, see online supplementary material). Benchmarking Universal Single-Copy Orthologs (BUSCO) analysis indicated that 99.0% of complete homologues were present in our assembled genome (Table S7, see online supplementary material). Furthermore, 117 telomeric repeat regions (TTTAGGG) were detected in our assembled 65 chromosomes, and 53 chromosomes harbored two telomeric regions at the beginning and end of the chromosomes (Table S8, see online supplementary material). Taken together, these results strongly support the high contiguity, accuracy, and completeness of our okra genome assembly.

**Table 1:** Summary of genome assembly and annotation

	*Abelmoschus esculentus*
Genome assembly	
Estimated genome size (Gb)	1.20
Assembled genome size (Gb)	1.19
Number of contigs	128
Contig N50 (Mb)	16.98
Chromosome numbers	65
Anchoring rate (%)	99.17
LAI	19.60
Genome annotation	
Repeat sequence	55.59%
Predicted gene models	113 364

**Figure 1:**
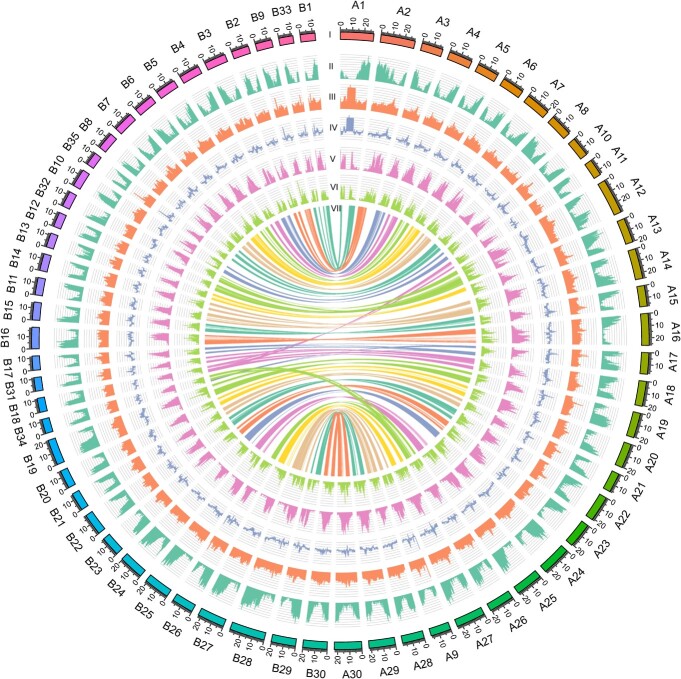
Overview of the *Abelmoschus esculentus* genome assembly. Circos representation of *A. esculentus* genome features. The outermost layer represents the 65 pseudo-chromosomes (scale mark = 1 Mb) (I). The density of protein-coding genes (II), GC content (III), GC skew (IV), LTR/*Gypsy* (V), and LTR/*Copia* (VI) were computed using a 200 kb non-overlapping window. The innermost track (VII) shows the synteny relationship between chromosomes, with colored curves displaying the inter-subgenome syntenic regions.

Interestingly, we observed an intra-genomic syntenic relationship of almost completely 1:1 in our assembled okra genome, and 15-mer enrichment analysis using SubPhaser showed that the 65 chromosomes could be clearly clustered into two independent groups (Fig. S4, see online supplementary material). Further analysis indicated that there existed great differences in the type and content of repeat sequences between the two sets of subgenomes (Figs S5 and S6, see online supplementary material). Thus, the okra genome was successfully phased into two subgenomes, with one group containing 30 chromosomes designated the A subgenome, and another group of 35 chromosomes designated the B subgenome (Fig. 1). Additionally, we identified the candidate centromeric regions in the 65 chromosomes (Fig. S7, see online supplementary material).

Overall, 55.59% of the *A. esculentus* genome sequence was identified as transposable elements (TEs). Notably, long terminal repeat retrotransposons (LTR-RTs) represented the most abundant class of repetitive sequences (Fig. S8, see online supplementary material), accounting for 32.26% of the *A. esculentus* genome. Among them, *Gypsy* elements (20.98%) comprised the majority of the LTR-RTs, followed by *Copia* elements (4.41%) (Table 1; Table S9, see online supplementary material). In total, 113 364 protein-coding genes (A: 57374; B: 55879) were predicted with high confidence (Table 1; Table S10, see online supplementary material), of which 95.89% could be functionally annotated by homologous sequences or protein domains (Table 1; Table S10, see online supplementary material). The average gene size and average exon number were 2480 bp and 5.23, respectively (Tables S10 and S11, see online supplementary material).

### Comparative genomics and gene family evolution analysis

**Figure 2:**
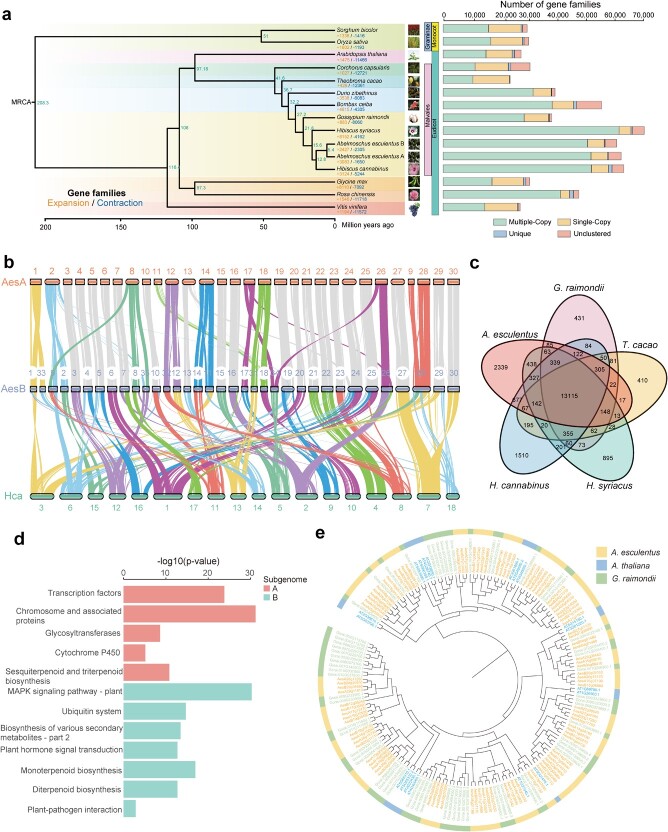
Comparative genomics analysis between *Abelmoschus esculentus* and neighboring species. **a** Phylogenomic analysis of *A. esculentus* with 13 other plant species. The species divergence times (marked in green at each node) were estimated using r8s. The gain and loss of gene families was analysed using CAFE. The number of gene families experiencing expansion and contraction are enumerated below the species names in orange and blue, respectively. Different categories of orthologous genes across all species are displayed as stacked bar charts on the right. **b** Synteny analysis between the two subgenomes of *A. esculentus* and *Hibiscus cannabinus*. Collinear analysis reflects the correspondence of chromosomes between the A and B subgenomes and chromosomal rearrangement events. For the chromosome pairs undergoing rearrangements, different colors were assigned for the syntenic blocks. **c** Venn diagram showing the numbers of common and specific gene families in each species. **d** KEGG enrichment analysis of gene families showing significant expansion in *A. esculentus* after divergence from the most recent common ancestor. **e** Phylogenetic tree of HD-Zip I subfamily members in *A. esculentus*, *G. raimondii*, and *Arabidopsis thaliana*.

To explore the evolution of okra, we constructed a species tree of 14 plants using 122 single-copy orthologous genes (Fig. 2a; Tables S12 and S13, see online supplementary material). The phylogenetic results showed that okra had the closest relationship with *H. cannabinus*, followed by *H. syriacus*. Okra and *H. cannabinus* diverged from their common ancestor 12.8 million years ago (Mya). The evolutionary relationship of representative Malvaceae species was revealed, and the divergence times corresponding to the *K*s peaks of gene pairs within syntenic blocks of these species agreed with those in the phylogenetic tree (Fig. 2a and c). The A and B subgenomes of okra diverged from each other at 5.4 Mya, and genome-wide synteny analysis of *H. cannabinus* and the okra A and B subgenomes showed that several fission and fusion events occurred between A and B subgenomes (Fig. 2b), which provides an explanation for the difference in chromosome number between the two subgenomes. Numerous chromosomal translocation events were also observed between them (Fig. 2b; Fig. S9, see online supplementary material).

Gene family analysis showed that 3083 and 2427 gene families were expanded in okra A and B subgenomes, respectively (Fig. 2a). WGD/segmental duplication represented the most abundant duplicate type among expanded gene families in both subgenomes (Fig. S10, see online supplementary material). Among them, 105 and 66 rapidly expanded gene families (*P*-value ≤0.01) were detected in the A and B subgenomes, respectively, and 2339 unique gene families were observed in the okra genome (Fig. 2d). Functional enrichment analysis indicated that the rapidly expanded gene families in A subgenome were mainly associated with Gene Ontology (GO) terms such as response to auxin (GO:0009733, *P* = 7.99 × 10^−6^), cell wall modification (GO:0042545, *P* = 6.68 × 10^−7^), the cellulose biosynthetic process (GO:0030244, *P* = 1.87 × 10^−7^), and cellulose synthase activity (GO:0016760, *P* = 1.87 × 10^−7^) (Table S14, see online supplementary material). They were significantly enriched in Kyoto Encyclopedia of Genes and Genomes (KEGG) terms such as plant hormone signal transduction (K14432, *P* = 5.88 × 10^−13^), isoflavonoid biosynthesis (K25075, *P* = 3.55 × 10^−14^), carbohydrate metabolism (K20628, *P* = 1.34 × 10^−6^), plant−pathogen interaction (K13457, *P* = 1.59 × 10^−6^), and terpene biosynthesis (K15803, *P* = 4.44 × 10^−9^) (Fig. 2e; Table S15, see online supplementary material). For the B subgenome, the rapidly expanded gene families showed over-representation of several GO terms, such as positive regulation of circadian rhythm (GO:0042753, *P* = 6.5 × 10^−25^), the respiratory electron transport chain (GO:0022904, *P* = 2.47 × 10^−9^), and cytochrome complex assembly (GO:0017004, *P* = 2.95 × 10^−8^) (Table S14, see online supplementary material), and were closely associated with KEGG terms such as starch and sucrose metabolism (K19891, *P* = 3.19 × 10^−24^), the MAPK signaling pathway (K14514, *P* = 4.07 × 10^−30^), and phenylpropanoid biosynthesis (K13065, *P* = 6.95 × 10^−13^) (Fig. 2e; Table S15, see online supplementary material). Collectively, the functional diversification between the A and B subgenomes may provide the genetic basis for the strengthened material synthesis ability and strong environmental adaptability in okra (Fig. 2e).

**Figure 3:**
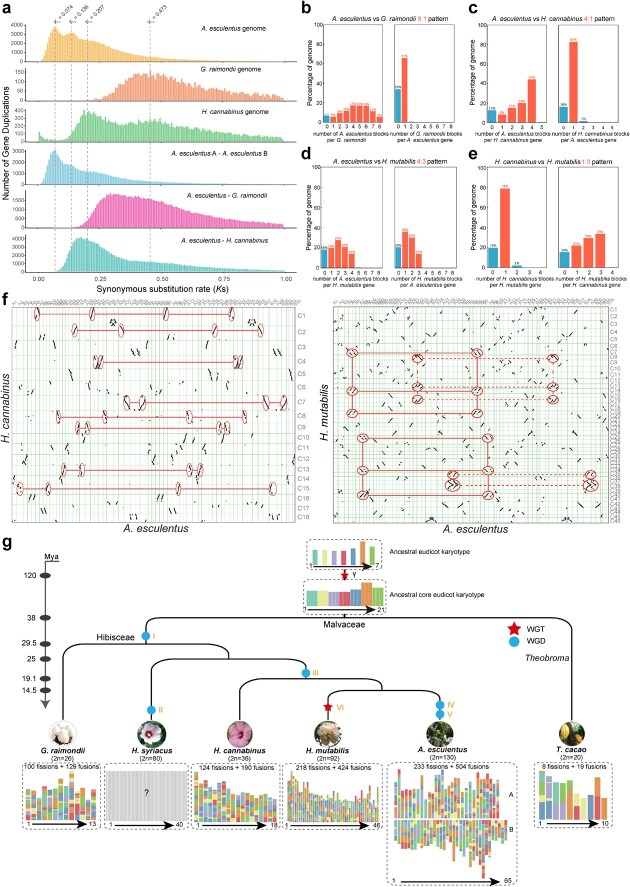
Widespread whole-genome duplication (WGD) and karyotype evolution in Malvaceae. **a** The histogram of inter- or intra-species synonymous substitution rates (*K*s) indicates whole-genome duplication (WGD) events and species divergence time. The four dashed lines indicate the positions of *K*s peaks in Malvaceae species. **b** Synteny depth ratio between *Abelmoschus esculentus* and *Gossypium raimondii*. **c** Synteny depth ratio between *A. esculentus* and *Hibiscus cannabinus*. **d** Synteny depth ratio between *A. esculentus* and *Hibiscus mutabilis*. **e** Synteny depth ratio between *H. cannabinus* and *H. mutabilis*. **f** Dot plot showing the syntenic relationship between *A. esculentus* and *H. cannabinus* (4:1, left panel), and *H. mutabilis* (4:3, right panel). **g** Chromosome evolution of *A. esculentus* and closely related species in the family Malvaceae based on the karyotypes inferred using WGDI. The inferred WGD events are indicated on the corresponding branches. The divergence times are displayed on the left side. WGD I was shared by the five Malvaceae species. WGD II occurred individually in *Hibiscus syriacus*. WGD III was shared by *H. cannabinus*, *H. mutabilis*, and *A. esculentus*. WGD IV and V represent two WGD events that were unique to *A. esculentus*. WGT VI was a triplication event that occurred individually in *H. mutabilis*. Blue ellipses indicate WGD events, and the red star represents the WGT event. The karyotype for *H. syriacus* is unknown (shown in grey) due to the lack of chromosome-level genome assembly.

We also reconstructed the phylogenetic tree with okra as an individual species to explore the changes to gene families in okra (Fig. S11, see online supplementary material). Interestingly, the expanded gene families in okra were significantly associated with transcription factors (K09338, *P* = 2.33 × 10^−14^) (Table S16, see online supplementary material), especially homeodomain-leucine zipper (HD-Zip) proteins. Previous studies showed that HD-Zip proteins participate in various physiological processes, such as abiotic stress responses, auxin signaling, controlling embryogenesis, anthocyanin accumulation, and root development [22–25]. Notably, the copy number of the HD-Zip family in okra (288) was much higher than in *Arabidopsis thaliana* (58) and *G. raimondii* (181) (Fig. S12 and Table S17, see online supplementary material), indicating that HD-Zip proteins have undergone remarkable amplification in okra. Of them, HD-Zip I had the most members (98), much greater than those in *A. thaliana* (19) and *G. raimondii* (64) (Fig. 2f). Because of the strong association of HD-Zip I with the response to hormone and abiotic stress, we hypothesized that the high copy number of this subfamily might contribute greatly to the response to abiotic stress by okra.

### Comprehensive phylogenetic analysis of the WGD events that occurred widely in Malvaceae species

To reveal the significant differences in chromosome number and genome size across the Malvaceae species, we inferred the WGD events that occurred in these species and identified three significant peaks at 0.074, 0.136, and 0.207 in okra (Fig. 3a). Meanwhile, a shared *K*s peak at 0.48 was observed in *G. raimondii* and *H. cannabinus*. This peak is older than the speciation peak between okra and each of *G. raimondii* and *H. cannabinus*, suggesting that the common ancestor of these three species commonly underwent this WGD event (named WGD I). Additionally, the same *K*s peak at 0.22 was also detected in the *H. cannabinus* genome (Fig. 3a), indicating a WGD event took place in the ancestry of okra and *H. cannabinus* (designated WGD III). Interestingly, two recent *K*s peaks were identified uniquely in the okra genome, implying that okra has independently undergone two rounds of WGD events. The *K*s peak of the orthologs between the two subgenomes of okra overlapped with the first *K*s peak of okra (Fig. 3a). Thus, the second *K*s peak indicates an independent WGD event in okra (named WGD IV), while the first *K*s peak actually represents a polyploidization event (named WGD V) in the okra genome.

To confirm the WGD events occurring in the Malvaceae species during evolution, we compared the syntenic depth ratios of the four different groups. First, the syntenic depth ratio between okra and *G. raimondii* displayed an 8:1 pattern (Fig. 3b), in support of the suggestion that okra has undergone three additional rounds of WGD events (Fig. 3b)*.* Second, the syntenic depth ratio for okra vs *H. cannabinus* was 4:1 (Fig. 3c), which was consistent with our previous hypothesis that okra have experienced two additional WGD events relative to *H. cannabinus*. The dot plot also displayed a clear syntenic depth ratio of 4:1 (Fig. 3f, left panel). Third, an overall 4:3 pattern was observed for okra and *H. mutabilis* (Fig. 3d), and a similar pattern was observed in the dot plot of okra vs *H. mutabilis* (Fig. 3f, right panel)*.* Three WGD events have previously been reported in *H. mutabilis* [19], and regarding the 4:3 ratio, we speculated that the most recent WGD reported in the *H. mutabilis* genome was a whole genome triplication (WGT) event that occurred independently after the divergence of okra and *H. mutabilis*. Fourth, we also calculated the syntenic depth ratio of *H. cannabinus* vs *H. mutabilis*, yielding an overall 1:3 pattern (Fig. 3e), further supporting an extra WGT occurring in *H. mutabilis* relative to *H. cannabinus*.

Based on the above inferred WGD events, a comprehensive phylogenetic tree of the Malvaceae species was constructed for the first time with the currently available genome assemblies (Fig. 3g). Notably, the chromosome numbers in Malvaceae species showed significant differences, ranging from *Theobroma cacao* (2n = 20) to okra (2n = 130). The WGD events that commonly and uniquely occurred in each species are displayed on the corresponding branches (Fig. 3g). In detail, *G. raimondii* only experienced one WGD event (WGD I), which is shared by all Malvaceae species. For *H. syriacus*, two WGD events were identified, one of which occurred individually in *H. syriacus* (named WGD II), as reported previously (Fig. 3g). *H. cannabinus* underwent two rounds of WGD events, including WGD I, shared by five Malvaceae species, and WGD III, shared by *H. cannabinus*, *H. mutabilis*, and okra. Remarkably, okra experienced two additional WGD events (WGD IV and WGD V) after splitting from *H. mutabilis*. *H. mutabilis* showed the closest relationship to okra, and it has experienced an independent WGT event (WGT VI) in addition to the two older WGDs (WGD I and III), which might partially explain the smaller difference in chromosome number between *H. mutabilis* and okra (Fig. 3g). Additionally, we inferred the karyotype of these Malvaceae species based on the ancestral eudicot karyotype (AEK) (Fig. 3g). Twenty-one ancestral chromosomes served as the basis for all core eudicot karyotype (ACEK) after the common ancestor of the core eudicots undergoing the γ-WGT event. Compared with the ACEK, the current *G. raimondii* genome arose from 100 genomic fissions and 129 genomic fusions; the *H. cannabinus* genome from 124 fissions and 190 fusions; the *H. mutabilis* genome from 218 fissions and 424 fusions; and the *A. esculentus* genome from 233 fissions and 504 fusions (Fig. 3g). It was also estimated that the *T. cacao* genome arose from eight fissions and 19 fusions. Apparently, the *T. cacao* genome is the most conserved among Malvaceae species, with the fewest chromosome shuffling events after the γ-WGT event, followed by the *G. raimondii* genome which is the most stable among Hibisceae species (Fig. 3g). By contrast, the okra genome underwent the most fissions and fusions (Fig. 3g), implying a large number of chromosome shuffling events occurring in okra, which is consistent with the number of WGDs experienced by these Malvaceae species. Altogether, these results revealed a comprehensive landscape of WGD events and karyotype evolution in Malvaceae species, suggesting that widespread WGD events and complicated chromosome evolution have occurred in Malvaceae species. This provides partial evidence to explain the significant differences in chromosome number across the Malvaceae species.

### Extensive genomic deletions and functional divergences between okra and *G. raimondii*

**Figure 4:**
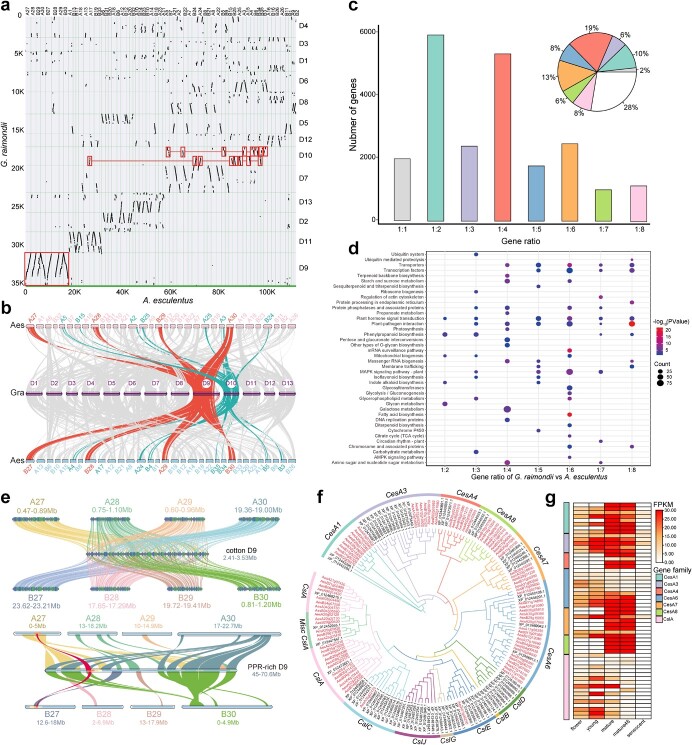
Huge deletion and functional differences revealed by comparison between *Abelmoschus esculentus* and *Gossypium raimondii.***a** Dot plot showing the synteny comparison between *A. esculentus* and *G. raimondii.* The colored blocks represent the most significant 8:1 syntenic relationship. **b** Syntenic comparison between *A. esculentus* and *G. raimondii* revealing the different syntenic pattern*.* The red lines represent a 1:8 syntenic relationship, the green lines represent a 1:16 syntenic relationship. **c** Bar plot showing the distribution of different gene ratios between *A. esculentus* and *G. raimondii*. The colored parts of the pie chart correspond to *A. esculentus* genes with different ratios, while the white part represents unique genes in *A. esculentus*. **d** KEGG enrichment analysis of *A. esculentus* genes showing different ratios (ranging from 1:2 to 1:8) against *G. raimondii*. **e** Microsynteny analysis of *G. raimondii* D9 and *A. esculentus* A27, A28, A29, A30, B27, B28, B29, and B30. Colored lines represent different *A. esculentus* chromosomes (top). Microsynteny analysis of *G. raimondii* D9 chromosome and eight *A. esculentus* chromosomes, and the red lines indicate the PPR-rich genomic regions (bottom). **f** Phylogenetic tree of the *CesA* and *Csl* subfamily genes in *G. raimondii* and *A. esculentus.* The *CesA* subfamily was divided into six clades, and the *Csl* subfamily was grouped into eight clades. **g** Expression pattern of *CesA* and *Csl* genes across five stages of *A. esculentus.* The colored rectangles on the left represent different clades in the phylogeny. The members of the *CesA* clades and the *CslA* clade are displayed in the heatmap because the members of other *Csl* clades had only weak or undetectable expression levels. To achieve a better heatmap effect, the FPKM value of genes with FPKM higher than 30 was uniformly set to 30 during the generation of the heatmap.

Despite the close evolutionary relationship between okra and *G. raimondii*, there are significant differences in the fruit morphology and polysaccharide types. First, the inter-genomic comparison showed a clear 8:1 syntenic relationship between okra and *G. raimondii* (Fig. 4a and b), further supporting the occurrence of three independent WGD events in okra after divergence from *G. raimondii*. What is puzzling is that although a clear 1:8 syntenic relationship was identified between *G. raimondii* and okra, their genome sizes (genome size ratio = 1:1.2) showed no significant difference (Fig. S13, see online supplementary material). Also, no obvious large syntenic regions were identified for the intra-genomic comparison of okra, except between the A and B homologous chromosomes (Figs S14 and S15, see online supplementary material). Therefore, we speculated that the okra genome may have undergone huge deletions during evolution after the WGD III and WGD IV events. To support this hypothesis, further comparison of the syntenic regions between okra and *G. raimondii* was conducted. The percentages of syntenic genes in *G. raimondii* with each of the eight okra chromosome groups were rather low, ranging from 12.45% to 26.90% (Fig. S16; Tables S18 and S19, see online supplementary material), suggesting that many genes homologous to cotton have been lost in the okra genome. Additionally, we obtained a set of non-syntenic cotton proteins (18 466/40976) and aligned them to the okra genome. The results showed that 58.93%–70.13% of the non-syntenic cotton proteins in D9 had no significant BLAT hit in the okra genome (Fig. S17, see online supplementary material), suggesting that the loss of syntenic genes can be attributed to the loss of genomic fragments in okra (Fig. S18, see online supplementary material).

We also summarized the numbers of gene copies retained after three WGD events in okra compared with *G. raimondii* (Table S20, see online supplementary material). Intriguingly, genes with ratios of 1:2, 1:4, and 1:6 between *G. raimondii* and okra showed remarkably high frequency (Fig. 4c), suggesting that large numbers of genes were retained in okra after WGD. KEGG enrichment analysis showed that these retained genes with different copy numbers after the WGDs were mainly enriched in material biosynthesis, signaling transduction, stress responses, and metabolism (Fig. 4d). Specifically, the genes with higher copy ratios of 6:1, 7:1, and 8:1 in okra compared with cotton were mainly enriched in plant–pathogen interaction (K13412, *P* = 8.43 × 10^−20^), the MAPK signaling pathway (K20717, *P* = 6.45 × 10^−5^), transcription factors (K09338, *P* = 1.58 × 10^−4^), diterpenoid biosynthesis (K04124, *P* = 0.023), and cytochrome P450 (K24184, *P* = 2.07 × 10^−6^) (Tables S21–S28, see online supplementary material). Taken together, these results indicate that the genes retained with multiple copy numbers in okra after WGD events were mainly related to the biosynthesis of secondary metabolites, which might contribute greatly to the resistance of okra against biotic and abiotic stress.

The D9 chromosome of *G. raimondii* was the most representative chromosome, showing synteny with eight almost complete okra chromosomes (Fig. S19, see online supplementary material), while several large deletions and rearrangements were detected (Fig. 4e). For example, a deletion at 51.02–52.68 Mb of the cotton D9 (GraD9) was detected on eight syntenic okra chromosomes, and we found that this region is rich in pentatricopeptide repeat (PPR) proteins. PPR genes are of interest because of their direct relation to cytoplasmic male sterility (CMS) in many crops, including *G. raimondii* [26]. Specifically, we identified a total of 57 PPR proteins in okra, which is much fewer than in *G. raimondii* (297) (Table S29, see online supplementary material), suggestive of the loss of most PPR genes during okra evolution and/or potential gene expansion in cotton domestication. The microsynteny relationship between the eight okra chromosomes and the PPR-rich genomic region of GraD9 showed that the PPR genes were mainly distributed on chromosomes A27 (11) and B27 (8), and there were more PPR genes in the *G. raimondii* region (36) than in okra (Fig. 4e, bottom; Fig. S20, see online supplementary material), which further supports extensive deletions of genomic segments in the okra genome.

Cotton is the main plant species for natural fiber production, in which cellulose synthesis-related gene superfamilies play important roles [27]. To elucidate the biological differences in polysaccharide types between okra and *G. raimondii*, we conducted a systematic analysis of the cellulose synthase (*CesA*) and cellulose synthase-like gene (*Csl*) subfamilies in okra and cotton. In total, 57 *CesA* genes were identified in okra genome, which is significantly more than the copy number in *G. raimondii* (30) (Fig. 4f). The *CesA* genes in okra were further classified into six clades, *CesA1*, *CesA3*, *CesA4*, *CesA6*, *CesA7*, and *CesA8* (Fig. 4f). Notably, the copy number in okra was higher than that in *G. raimondii* for almost all clades except for *CesA3* (Table S30, see online supplementary material). For the *Csl* subfamily, we observed an opposite trend in the copy number between okra and *G. raimondii*, and the total copy number of the *Csl* genes in okra (27) was much lower than in *G. raimondii* (48) (Table S30, see online supplementary material). However, the *CslA* clade was significantly expanded in okra (20) compared with *G. raimondii* (3) (Fisher’s exact test, *P* < 1 × 10^−3^). For the *CslC* and *CslE* clades, only one copy of each was identified in okra while 12 and 9 genes, respectively, were identified in cotton. Interestingly, no members of the *CslB*, *CslD*, *CslJ*, and *CslG* clades were detected in okra. The *CesA* subfamily is mainly related to cell wall formation and reinforcement, and the *Csl* gene family is the major participant in cellulose synthesis. Gene expression analysis (Table S31, see online supplementary material) indicated that the majority of the *CesA* genes were highly expressed in both young and ripe pods in okra (Fig. 4g). However, 78% of the *Csl* genes showed no or weak expression levels. Altogether, our findings revealed that the vast differences in copy number of the *CesA* and *Csl* subfamilies between okra and *G. raimondii* might partially explain the significant differences in the type and amount of fiber production between the two species.

### Insights into the biosynthesis pathway of sinapic acid/S-lignin in okra

**Figure 5:**
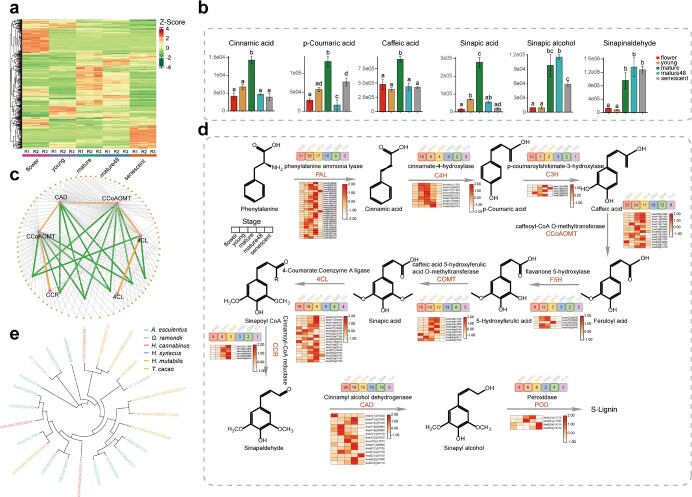
Insights into the biosynthesis pathway of sinapic acid and S-lignin in okra based on transcriptome and metabolome data. **a** Heatmap displaying the content of secondary metabolites identified in different okra samples by untargeted metabolite profiling. **b** Gene co-expression subnetwork module significantly related to sinapic acid content. The orange nodes represent five key enzymes participating in S-lignin biosynthesis in this subnetwork, and the green lines represent genes with significant associations with these enzyme genes. **c** The content of metabolites in the S-lignin biosynthesis pathway detected in different tissues. Different letters above columns represent a significant difference between the samples examined. Statistical test was carried out using one-way ANOVA (analysis of variance). **d** The inferred S-lignin biosynthesis pathway in *Abelmoschus esculentus.* The numbers above each gene symbol represent the copy number in the corresponding species. The heatmap displays the transcript levels of enzyme-encoding genes in this pathway across the five stages. **e** Phylogenetic tree of *CCR* enzyme genes in *A. esculentus, Gossypium raimondii, Hibiscus syriacus, Hibiscus cannabinus*, *Hibiscus mutabilis*, and *Theobroma cacao.*

To reveal the main active components of okra and their underlying biosynthesis mechanism, we performed transcriptome and metabolome analysis of the okra samples collected at different developmental stages, including flowers, young pods, mature pods, mature pods that lay at 20°C for 48 hours (hereafter referred to as mature48 pods), and senescent pods. Metabolomics analysis showed that 589 compounds were identified in these samples in okra (Fig. S21, see online supplementary material). Among them, flavonoids were the most plentiful compounds, occupying 33.11% of all metabolites, followed by phenolic acids and alkaloids, comprising 25.81% and 15.45%, respectively (Fig. S22, see online supplementary material). Hierarchical clustering analysis indicated that the metabolite contents displayed obvious stage/organ-specific patterns (Fig. 5a). Based on transcriptome analysis across these samples, we identified 37 493 differentially expressed genes (DEGs) (Fig. S23, Table S32, see online supplementary material), which were further divided into eight clusters with distinct expression patterns. As the mature pod of okra is the main part consumed as food, we further analysed the genes with high expression only in mature pods (Fig. S24, see online supplementary material). Functional enrichment analysis (Fig. S25, see online supplementary material) indicated that these genes were mainly associated with the biosynthesis of various plant secondary metabolites, including phenylpropanoid biosynthesis (K00430, *P* = 6.50 × 10^−4^) and phenylalanine metabolism (K13064, *P* = 9.38 × 10^−5^), suggesting the highly active status of material synthesis in mature pods.

Our metabolome analysis showed that phenolic acids were one of the most abundant compounds in okra. Sinapic acid is one of the key phenolic acids as it is beneficial to multiple aspects of human health, with antibacterial, anticancer, anti-inflammatory, and anti-oxidant effects. The sinapic acid content was highest in mature pods and exhibited the most significant changes between samples from distinct developmental stages (Fig. 5b). A gene co-expression network was built based on the DEGs through weighted correlation network analysis (WGCNA). The results showed that the MEgreen module, containing 2306 genes, showed significant correlation with the phenolic acid content (Figs S26 and S27, see online supplementary material). To further clarify the relationship between the DEGs and compounds related to the sinapic acid/S-lignin biosynthesis pathway, the genes in the MEgreen module were filtered to obtain a co-expression subnetwork based on the FPKM and edge weights (Table S33, see online supplementary material). As illustrated in Fig. 5c, a tiny subnetwork comprising six enzyme-encoding genes (two 4CL, two CCoAOMT, one CAD, and one CCR gene) involved in sinapic acid/S-lignin biosynthesis was obtained, of which four hub genes displayed dense connectivity with other peripheral genes. Interestingly, these peripheral genes were mainly over-represented in the cellulose biosynthetic process (GO:0030244, *P* = 1.54 × 10^−7^), cell wall macromolecule catabolic process (GO:0016998, *P* = 0.02), protein glycosylation (GO:0006486, *P* = 0.01), and the chitin catabolic process (GO:0006032, *P* = 1.54 × 10^−7^) (Fig. S28, see online supplementary material). Additionally, five transcription factors (one HB, two WRKY, one AUX/IAA, and one Trihelix) were identified in the peripheral genes (Table S34, see online supplementary material), suggesting their potential regulatory roles in the expression of these hub genes. We also explored the regulatory network of the gene families related to the biosynthesis of sinapyl alcohol, which plays an essential role in S-lignin biosynthesis (Figs S29 and S30, see online supplementary material).

To further explore the reason for the different sinapic acid content between different samples, we identified the major enzyme genes participating in the biosynthesis pathway. In general, the expression patterns of the enzyme genes almost entirely followed the trend of the compound content. Most of the enzyme-encoding genes showed a tendency to be highly expressed in young and mature pods during the process of nutrient accumulation, especially *C4H*, which plays a leading role in the catalysis of cinnamic acid to p-coumaric acid. Notably, in mature pods, there was a much higher content of p-coumaric acid than cinnamic acid (Fig. 5b), which follows the expression patterns of the related enzyme genes. In the steps following p-coumaric acid, most of the enzyme-encoding genes showed high expression in mature and mature48 pods. For example, COMT directly catalyzes 5-hydroxyferulic acid into sinapic acid, and most of the *COMT* genes displayed high expression levels in mature and mature48 pods. As a key enzyme in sinapaldehyde synthesis, CCR catalyzes sinapoyl-CoA to sinapaldehyde in the S-lignin biosynthesis pathway. Strikingly, we found that all the genes homologous to *CCR* in okra were highly expressed in mature48 pods (Fig. 5d), suggesting the onset of the lignification process in mature pods after harvest. Most compounds were maintained at a lower content in senescent pods; however, the content of sinapaldehyde increased significantly in senescent pods (Fig. 5b), consistent with the high expression patterns of the *CCR* genes. Sinapaldehyde is the second-last intermediate metabolite in the S-lignin synthesis pathway. An increase in its content indicates active lignin synthesis, which is consistent with the physiological changes of okra pods (from young to lignified). POD catalyzes the final reaction step of S-lignin synthesis. Interestingly, we found that two homologous *POD* genes (*AesB29g14210* and *AesB29g14230*) were significantly highly expressed in senescent pods relative to other stages (Fig. 5d), suggestive of the accelerated lignification process in senescent pods, as reported previously [28].

Additionally, we compared the homologous genes involved in the sinapic acid/lignin synthesis pathway between okra and five other closely related species. Generally, the copy numbers of almost all the enzyme-encoding genes (such as *C4H*, *C3H*, *COMT*, *CAD*, and *POD*) were significantly higher in okra than in other species, especially *COMT* and *CCR* (Fig. 5e). Considering the high ornamental values of *H. syriacus, H. cannabinus*, and *H. mutabilis*, and the important status of *G. raimondii* in natural fiber production, we speculated that the significantly increased copy number in okra of the gene families related to the production of secondary metabolites, such as sinapic acid, underlies the biological characteristics of okra, such as high nutritional value.

## Discussion

A growing number of studies have confirmed the great potential of okra in the prevention and alleviation of various diseases and in strengthening the immune system. However, the lack of a high-quality okra genome has substantially hampered further research to clarify the underlying biosynthetic mechanisms of the active components. Herein, we assembled a chromosome-level genome for this important vegetable crop. Many okra tissues contain a high quantity of gelatinous substances such as pectin, substantially increasing the difficulty of extracting the high molecular-weight genomic DNA that is required for long-read sequencing and seriously hindering the pace of decoding the okra genome. Undoubtedly, the achievement of assembling the okra genome will not only gain insights into the evolution of major Malvaceae species, but also facilitate the functional study and improvement of okra varieties.

Previously, there have been controversies about the chromosome number in the okra genome, and the reported okra chromosome number has ranged from 2n = 72 to 2n = 130, based on karyotype analysis [21, 29]. In our study, the chromosome number of okra was determined as 2n = 130 by FISH-based karyotype analysis and Hi-C data-assisted chromosome anchoring, which was consistent with previous reports [30, 31]. Additionally, a previous study indicated that okra might be a polyploid species [30], which was validated by our genome assembly and subgenome phasing. Based on the k-mer profile, the 65 assembled okra chromosomes were categorized into two distinct groups with unequal chromosome numbers (one with 30 chromosomes, and the other with 35 chromosomes). Intriguingly, the subgenome grouping showed a similar result to the inference of subgenome sources in a previous study, in which it was proposed that the modern okra chromosome number was likely to be derived from the hybridization of a 29-chromosome ancestor species (donor species A) and another ancestor species with 36 chromosomes (donor species B) [30]. To date, *Abelmoschus tuberculatus* is the only reported species with n = 29 chromosomes, which might be the putative donor species A. At least three Malvaceae species have n = 36 chromosomes, *Abelmoschus ficulneus* [32], *Abelmoschus moschatus* [21], and *Hibiscus esculentus* [29], thus making it a challenging task to infer the potential donor species B.

The evolutionary relationships among the Malvaceae species were first resolved, suggesting that okra is closest phylogenetically to species in the genus *Hibiscus*. Additionally, we found that widespread WGD events were detected in the Malvaceae species, leading to great differences in chromosome number and ploidy level. Notably, okra underwent three additional WGD events compared with diploid cotton. WGD events can provide rich genetic resources allowing strong environmental adaptation through neofunctionalization and/or subfunctionalization of duplicated genes. For example, more copies of genes related to plant–pathogen interactions were identified in okra compared with *G. raimondii* (Fig. 4d), such as WRKY transcription factors, potentially enabling okra to possess stronger environmental adaptability and disease resistance. Strikingly, there is a sharp contrast between the large gene number and the small genome size in okra. Intriguingly, our comparative analysis indicated that the okra genome underwent large genomic deletions and gene loss after three WGD events; however, many gene copies related to metabolite biosynthesis were preferentially retained, which possibly correlates with the enhanced environmental adaptability and abundant nutrient content of okra.

Additionally, a previous study reported two additional WGD events occurring in the *H. mutabilis* genome [19]. Interestingly, our results indicate that the most recent WGD event in *H. mutabilis* was actually a WGT event (Fig. 3d and f). This result is further supported by the recently reported *H. hamabo* genome (n = 46), indicating that *H. hamabo* experienced a WGD event and a WGT event after divergence from *G. raimondii* [20]. The reinterpretation of the recent WGD event in *H. mutabilis* as a WGT event corroborates the importance of abundant genome assemblies for comprehensive comparative analysis between Malvaceae species.

There are a variety of bioactive constituents in okra with widespread bioactive effects. In this study, a catalogue of 589 metabolites was identified at different developmental stages, of which flavonoids and phenolic acids represented the top two most abundant classes of compounds in okra. The phenolic acid content change between different developmental stages showed high correlation with the expression of sinapic acid biosynthesis-related genes. Thus, we focused on the biosynthesis pathway of sinapic acid and S-lignin and revealed the tendency of change of the intermediate metabolites and the expression pattern of candidate genes participating in this pathway. Okra is rich in edible dietary fibers, which might be strongly associated with the expansion of the *CesA* family in the okra genome. Additionally, the hardening process of okra pods after harvest has been mainly attributed to the reduction of cellulase activity and the accumulation of lignin. In this study, the nutrient content change and pod senescence was linked with the regulatory network of gene expression through a combination of transcriptomic and metabolomic analysis.

## Materials and methods

### Plant materials, DNA and RNA sample collection, and sequencing

The seeds of the okra cultivar ‘Wufu’ were purchased from the shop and planted in Hebei University. For genome sequencing, the leaves of fresh plants using a DNA extraction kit were used for extraction of high-molecular-weight genomic DNA. Then, two PCR-free SMRTbell libraries were constructed and sequenced on PacBio Sequel II (Annoroad Gene Technology Co., Ltd, Beijing, China) to yield sequencing data. For Hi-C sequencing, formaldehyde was used for crosslinking the fresh leaves, and the crosslinking reaction was terminated using glycine solution. Subsequently, the Hi-C library was built based on the instructions and sequenced on the Illumina platform (Annoroad Gene Technology Co., Ltd). For transcriptome sequencing, the aboveground and underground parts of seedlings were collected. These tissue samples were rinsed using ddH_2_O and stored at −80°C until use after snap-freeze using liquid nitrogen. Each sample includes three biological replicates. Total RNA extraction was performed using the RNeasy Plant Mini Kit (Qiagen, Hilden, Germany). A cDNA library was built following the instructions, followed by paired-end sequencing on the NovaSeq platform (Illumina).

### Genome assembly and quality assessment

To evaluate the genome feature of *A. esculentus*, k-mer frequency analysis was conducted using GCE (v1.0.2) [33] with default parameters. To achieve high-quality genome assembly, high-fidelity (HiFi) reads were obtained from the PacBio Sequel II platform. First, CANU (v2) was used for *de novo* genome assembly of *A. esculentus* with default parameters. Then, these contigs were scaffolded onto chromosomes using Hi-C reads. The assembled contigs were anchored onto chromosome pseudomolecules through sorting, orientation, and ordering using 3D-DNA (v170123) [34] and Juicer (v1.6) [35], yielding a final version of the *A. esculentus* genome assembly. The assessment of genome assembly quality was conducted using BUSCO (v4.1.4) [36] based on the embryophyta_odb10 dataset (containing 1614 core genes across land plants) and the LTR assembly index (LAI) [37].

### Genome annotation

The repetitive elements in the *A. esculentus* genome were annotated with *de novo* and homology-based approaches. TRF (Tandem Repeat Finder, v4.07b) [38] was used to identify tandem repeat sequences. For *de novo* searches, RepeatModeler (v1.0.11), LTR_FINDER (v1.05) [39], LTRharvest (v1.5.11) [40], and LTR_retriever (v1.9) [41] were applied for constructing *de novo* repeat libraries, and RepeatMasker (v4.1.1) was employed to detect repeat sequences using the *de novo* library. For homology-based searches, repeat elements were identified by RepeatMasker using a known repeat library (Repbase 15.02). Additionally, tRNAscan-SE [42] software was used for predicting tRNA genes. Other non-coding RNAs (e.g., rRNA, miRNA, and snRNA) were predicted using INFERNAL (v1.1) [43] software through search against the Rfam database (v9.1) [44].

To annotate coding genes in the *A. esculentus* genome, gene models were obtained by combining three approaches. For *ab initio* prediction, PASA (v2.3) [45] was performed to predict gene structure using transcripts assembled by Trinity (v2.12) [46], which were further used for gene model training in AUGUSTUS (v3.2.3) [47]. For homology-based prediction, the GenomeThreader (v1.7.3) [48] program was used for search against the protein sequences of seven Malvaceae species. For transcriptome-based prediction, Trinity was used for assembling transcripts based on RNA-seq data, and PASA software was used for gene structure prediction based on transcriptome assembly. Additionally, HISAT2 (v2.2.1) [49] was employed for RNA-seq reads mapping onto the genome, and StringTie (v2.1.6) [50] was used for generation of transcript structure. The assembled transcripts were subsequently used for ORF (open reading frame) prediction using TransDecoder (v5.1.0). To obtain the final gene set, EVidenceModeler (EVM) [51] were employed for combining prediction results from different sources. BUSCO was used for evaluating the annotated gene set based on the embryophyta_odb10 dataset.

### Subgenome division of *A. esculentus* genome

To divide the *A. esculentus* subgenomes, subgenome-specific repetitive DNA sequences were identified using SubPhaser [52]. Jellyfish v2.2.10 [53] was used to scan and count 15-mers on the 65 chromosomes. After normalization of the differential k-mer matrices, the chromosomes were clustered into two groups (subgenomes) using the k-means algorithm.

### Functional annotation

To predict the potential function of the annotated gene models in the *A. esculentus* genome, we performed a BLAST search of protein-coding genes against the UniProtKB/SwissProt protein database. Additionally, GO assignment and Pfam domain annotation were performed by InterProScan (v5.32) [54, 55]. KEGG orthology (KO) terms for each gene were assigned by homology searches against the hidden Markov model (KOfam) database using KofamScan [56] with the default parameters.

### Species tree construction and estimation of evolutionary rate

Orthologous genes in two subgenomes of *A. esculentus* and 13 other plants, including *A. thaliana*, *Corchorus capsularis*, *T. cacao*, *Durio zibethinus*, *Bombax ceiba*, *G. raimondii*, *H. syriacus*, *H. cannabinus*, *Glycine max*, *Rosa chinensis*, *Vitis vinifera*, and two outgroup species, *Sorghum bicolor* and *Oryza sativa* were assigned by using the OrthoFinder (v2.3.14) pipeline [57]. A maximum likelihood tree was built by RAxML (v2.1.9) [58] based on single-copy genes. The calibration of species divergence time was conducted using r8s [59] based on the TimeTree website [60]. FigTree (v1.4.3) was used for visualization of the species tree.

### Gene family analysis

CAFE v4.0.1 [61] was employed for analysing the change of gene families with the default parameters. We selected the gene families that were present in at least five species for further analysis. Generally, the changes in gene families of each lineage was evaluated using the built-in model. The change of gene families was analysed for all nodes and species.

### Whole-genome duplication, synteny analysis, and karyotype inference

Synonymous substitution rate (*K*s) estimation was used for detecting whole-genome duplication (WGD) in *A. esculentus*. First, the protein sequences of *A. esculentus*, *G. raimondii*, *H. cannabinus*, and *H. syriacus* were self-aligned using BLASTP with an E-value of 1 × 10^−5^. Then, MCScanX [62] was used for identifying the collinear blocks in these plants. KaKs_calculator (v2.0) [63] was employed for calculating *K*s values of homologous gene pairs with the YN model. The putative WGD events within the Malvaceae species were identified based on *K*s distributions of all gene pairs. To explore the karyotype evolution of Malvaceae species, we inferred the karyotypes of five Malvaceae species using the same method as described previously [64]. Briefly, the karyotypes of the current Malvaceae species were constructed through the –km and –k subroutines of WGDI [65] based on the proteins of the ancestral core eudicot karyotype (ACEK). The number of fission and fusion events occurring in each species was also calculated as described previously [64].

### Identification of HD-zip genes and phylogenetic analysis

We used iTAK [66] to identify transcription factors from protein sequences. The HD-Zip family members in *G. raimondii* and *A. thaliana* were retrieved from PlnTFDB v3.0 [67]. The HD-Zip protein sequences were aligned with MAFFT [68], and the best model was estimated by IQ-TREE 2 [69]. The online tool iTOL [70] was used for tree visualization.

### Comparison of syntenic relationship between *A. esculentus* and *G. raimondii*

A syntenic dot plot between *A. esculentus* and *G. raimondii* was generated using JCVI (v1.2.1) [71] with the default parameters. The comparison of chromosomes between *A. esculentus* and *G. raimondii* was generated using MCScan [62, 71] with the default options.

### Identification of the PPR-P subfamily in the *A. esculentus* genome

A BLASTp search was performed to identify PPR-P homologous genes in *A. esculentus* based on the PPR-P genes reported in *G. raimondii* with an E-value of 1 × 10^−5^. HMMER [72] was used to identify the conserved protein domain (Pfam accession ID ‘PF13041.1’). The chromosomal distribution of PPR-P genes was plotted using the genomicDensity package.

### Identification of the *CesA* and *Csl* families in *A. esculentus* and phylogenetic analysis

To identify the *CesA* and *Csl* families in *A. esculentus*, the *CesA* and *Csl* genes in *G. raimondii* were used as the query in a BLASTP search of *A. esculentus* protein sequences with an E-value of 1 × 10^−5^ [27]. HMMER [72] was also used to identify the conserved CesA (PF03552.9) and Csl (PF13641.1) protein domains. The protein sequences of the Ces superfamily members were aligned with MAFFT [68], and the best model was estimated by IQ-TREE 2 [69]. The online tool iTOL [70] was used for the visualization of the phylogenetic tree.

### Transcriptome analysis

To gain insight into the expression profiles across different okra stages, we collected tissue samples from five stages, including flowers, young pods, mature pods, mature pods that lay at 20°C for 48 hours (hereafter referred to as mature48 pods), and senescent pods, and each tissue including three replicates. After total RNA extraction for each tissue sample, a cDNA library was prepared following the instructions and sequenced on the Illumina platform (Annoroad Gene Technology Co, Ltd.). Raw reads were preprocessed using fastp (https://github.com/OpenGene/fastp) [73] to trim adapters and filter low-quality and short reads. For each tissue, HISAT2 [49] were used for mapping reads onto the reference genome. StringTie [50] was used for estimating the gene expression levels, measured as FPKM values. The DESeq2 package [74] was used for identifying DEGs between different tissues. The DEGs between different tissues should meet the following requirements: log_2_(fold change) > 2 and adjusted *P*-value <0.01, yielding a total of 37 493 DEGs. The 37 493 DEGs were divided into eight clusters using the Mfuzz package [75], with each cluster representing an expression pattern.

### S-lignin biosynthesis pathway analysis

We used BLASTP to search for the enzyme-encoding genes participating in the S-lignin pathway (E-value = 1 × 10^−5^). HMMER [72] was used to predict the conserved protein domains based on the reference sequences. We used TBtools [76] to create a heatmap of the FPKMs (normalized by column) of enzyme genes.

### Preparation of metabolic samples

Okra tissue samples were extracted using the method as described previously [77]. Briefly, samples were freeze-dried and a mixer mill was used for grinding at 30 Hz for 90 sec. Subsequently, 1.2 ml methanol solution (methanol:H_2_O, 70:30, v/v) was used to dissolve the lyophilized powder, vortex mixed for 0.5 min every half an hour and repeat for six times, finally centrifuged at 12000 rpm for 3 min. Prior to UPLC-MS/MS analysis, the supernatants were collected and filtrated as described previously [77]. To test the instrument stability, multiple samples for quality control were obtained by equally mixing the samples as described previously [78].

### Metabolome analysis

For metabolome analysis, an ultra-high-performance liquid chromatography–tandem mass spectrometry (UPLC-MS/MS)-based non-targeted metabolic profiling method [77, 79, 80] was applied for analysing the samples. Non-targeted metabolome analysis was conducted as described previously [77]. The composition and gradient program of mobile phase was set as described previously [77]. The other parameters of liquid chromatography were set as described previously [78]. Data was acquired based on the settings as described previously [77]. Additionally, the parameters for instrument tuning and mass calibration were set as described previously [78]. MRM experiments were performed as described previously [77]. The Metware database (MWDB) (Metware Biotechnology Co., Ltd, Wuhan, China) was used for identification of secondary metabolites. The Analyst v1.6.3 and Multiquant v3.0.2 were employed for processing and quantifying mass spectrometry data.

### Gene co-expression network analysis

For constructing a co-expression network, we used the 15 RNA-seq datasets from five different tissues. We performed WGCNA [81] based on the identified DEGs through the WGCNA package using Pearson correlations. We also related the metabolite content to module eigengenes and screened the modules most correlated with sinapic acid and sinapyl alcohol through clustering analysis between the modules and target compounds (sinapic acid, MEgreen; sinapyl alcohol, MEblue). For the sinapic acid co-expression network, we first selected the genes with an average FPKM value >2 for further screening. Then, we filtered gene pairs based on the weights between them, retaining gene pairs with weights >0.3.

For the sinapyl alcohol co-expression network, we found that the MEblue module contained a large number of transcription factor-related genes. Similarly, we filtered the module genes and retained gene pairs with weights >0.8. The co-expression network was imported into Cytoscape [82] for visualization.

## Acknowledgments

This work was supported by the Natural Science Foundation of Hebei Province (Grant No. C2021201048) and the Interdisciplinary Research Program of Natural Science of Hebei University (Grant No. 513201422004). We thank Catherine Perfect, MA (Cantab), from Liwen Bianji (Edanz) (www.liwenbianji.cn), for editing the English text of a draft of this manuscript.

## Author contributions

H.D. and L.X. conceived and supervised the project; L.X. and H.D. designed the paper; L.X., R.W., and H.D. wrote the paper; R.W., W.L., Q.H., H.Z., and X.Z. sequenced and processed the raw data; R.W., L.X., W.L., Q.H., and Z.L. assembled and annotated the genome; L.X., R.W., and Y.W. performed the phylogenetic and genome evolution analysis; R.W., L.X., and C.D. analysed the metabolome data; R.W., L.X., M.W., and W.L. conducted the transcriptome analysis.

## Data availability

The whole genome sequencing data reported in this study have been deposited in the Genome Warehouse in the National Genomics Data Center [83, 84], Beijing Institute of Genomics, Chinese Academy of Sciences/China National Center for Bioinformation, under accession number GWHBWBG00000000, and are publicly accessible at https://ngdc.cncb.ac.cn/gwh.

## Conflict of interest statement

The authors have declared no competing interests.

## Supplementary data

Supplementary data is available at *Horticulture Research* online.

## Supplementary Material

Web_Material_uhad120
