# Supplementary material for: The genome of okra (Abelmoschus esculentus) provides insights into its genome evolution and high nutrient content
Source: Hortic Res. 2023 Jun 2;10(8):uhad120. doi: 10.1093/hr/uhad120 (PMC10405168; doi:10.1093/hr/uhad120)
Supplement: Web_Material_uhad120 [file web_material_uhad120.zip › Supplementary Figure-20230505.pdf]

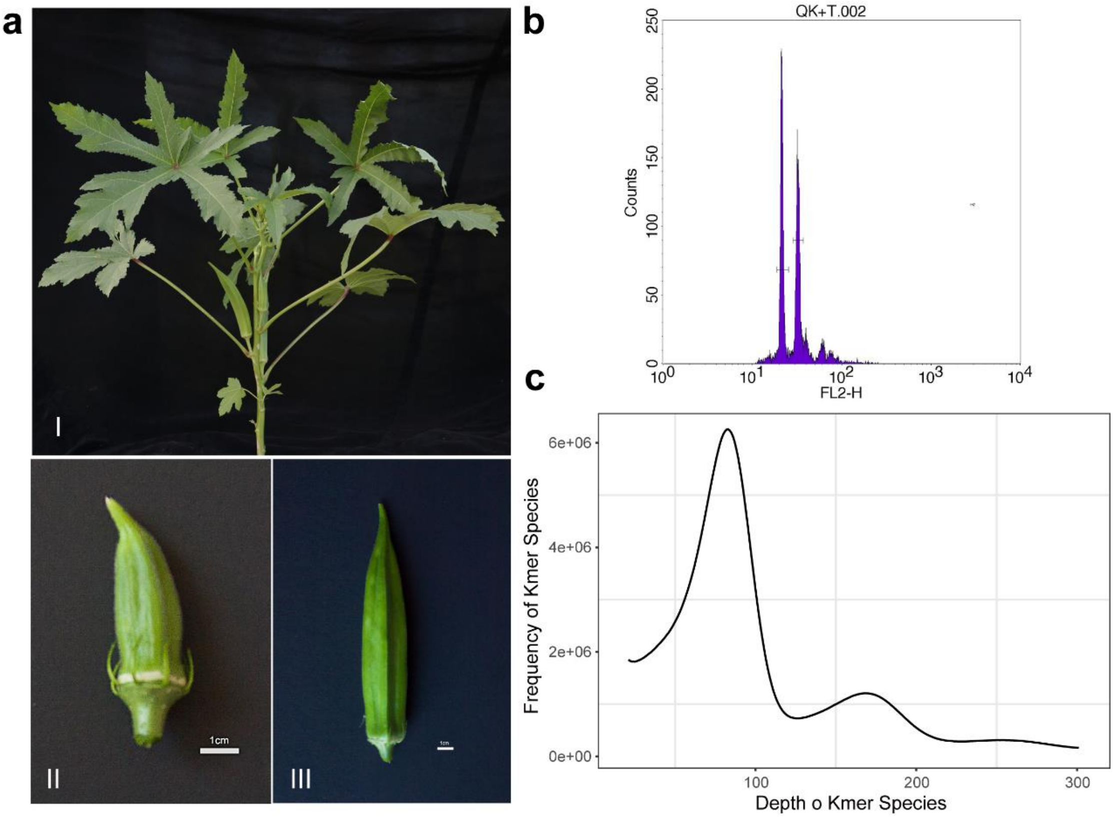

Supplementary Figure 1 Morphological characteristics and estimation of *A. esculentus* genome size. **(a)** Morphological characteristics of different tissues/parts of of *A. esculentus*, including the whole plant, flowers and pods. Scale bar, 1 cm. **(b)** Genome size estimated by flow cytometry (tomato and maize were used as the internal standard). **(c)** 17 k-mer survey result of *A. esculentus* genome. The main peak corresponded to the depth about 83, the genome size was estimated as 1.2 Gb, and the heterozygosity was 0.6%.

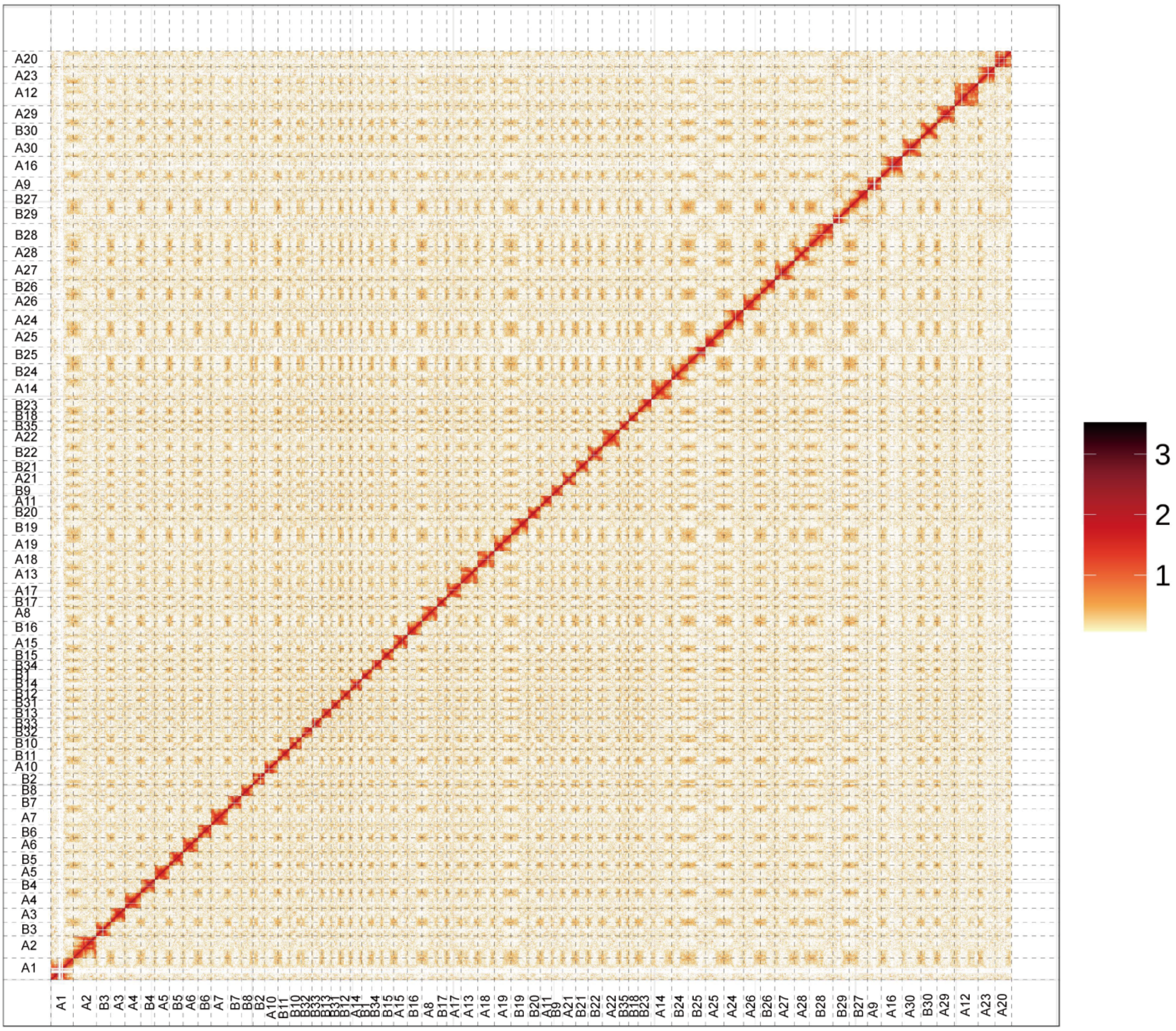

Supplementary Figure 2 Genome-wide Hi-C interaction map of *A. esculentus*. The heat map shows the intensity signal of Hi-C chromosome interaction.

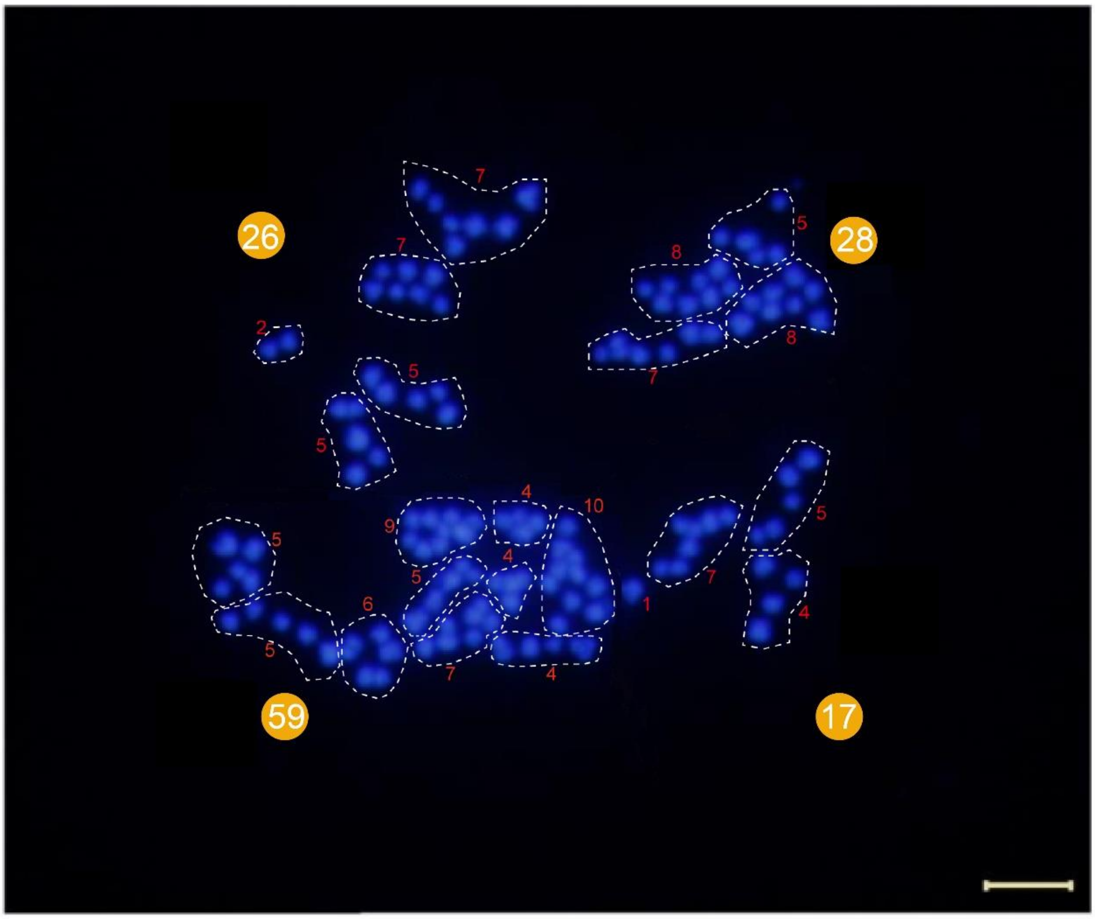

Supplementary Figure 3 Karyotype analysis of the *A. esculentus* genome based on fluorescence *in situ* hybridization (FISH) imaging using the root tip meristem.

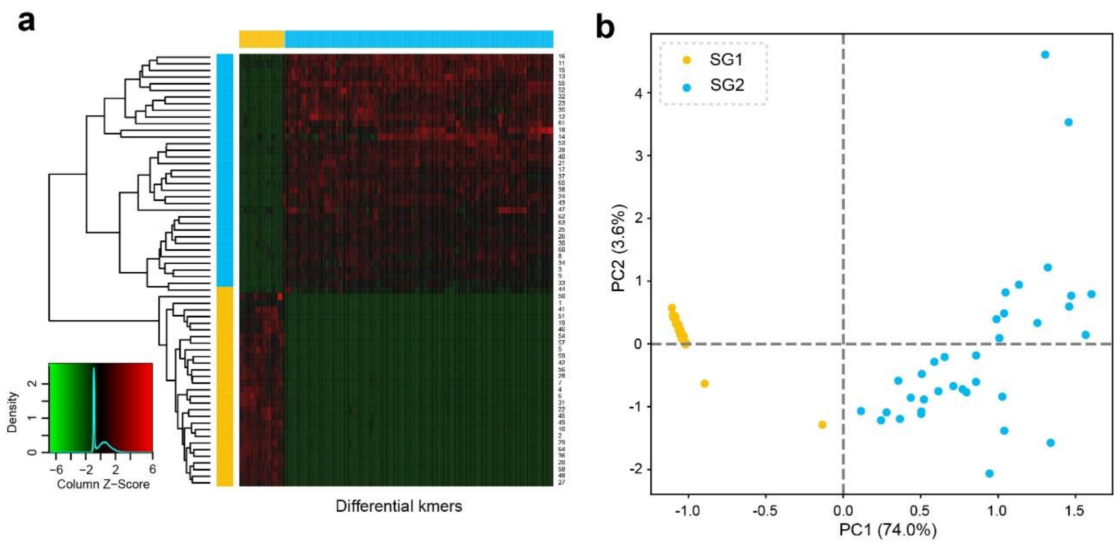

Supplementary Figure 4 SubPhaser analysis of the genome sequence of *A. esculentus*. (a) Clustering heatmap of differential 15-kmers between two subgenomes. **(b)** PCA of chromosomes in the okra genome based on 15-kmers.

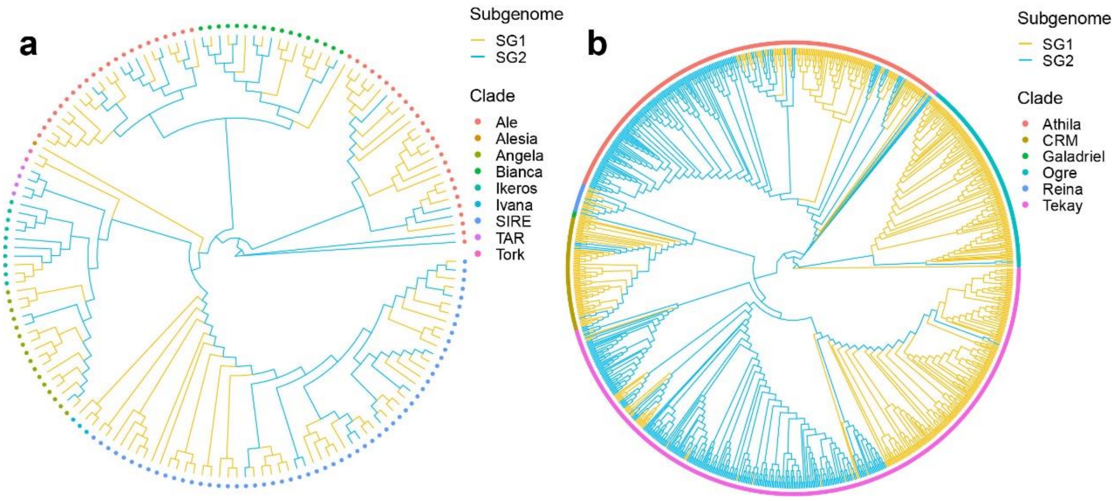

Supplementary Figure 5 Phylogenetic tree of LTR subclasses identified in the A and B subgenomes of *A. esculentus*. (a) Phylogenetic tree of Copia-type LTRs in *A. esculentus*. **(b)** Phylogenetic tree of Gypsy-type LTRs in *A. esculentus*.

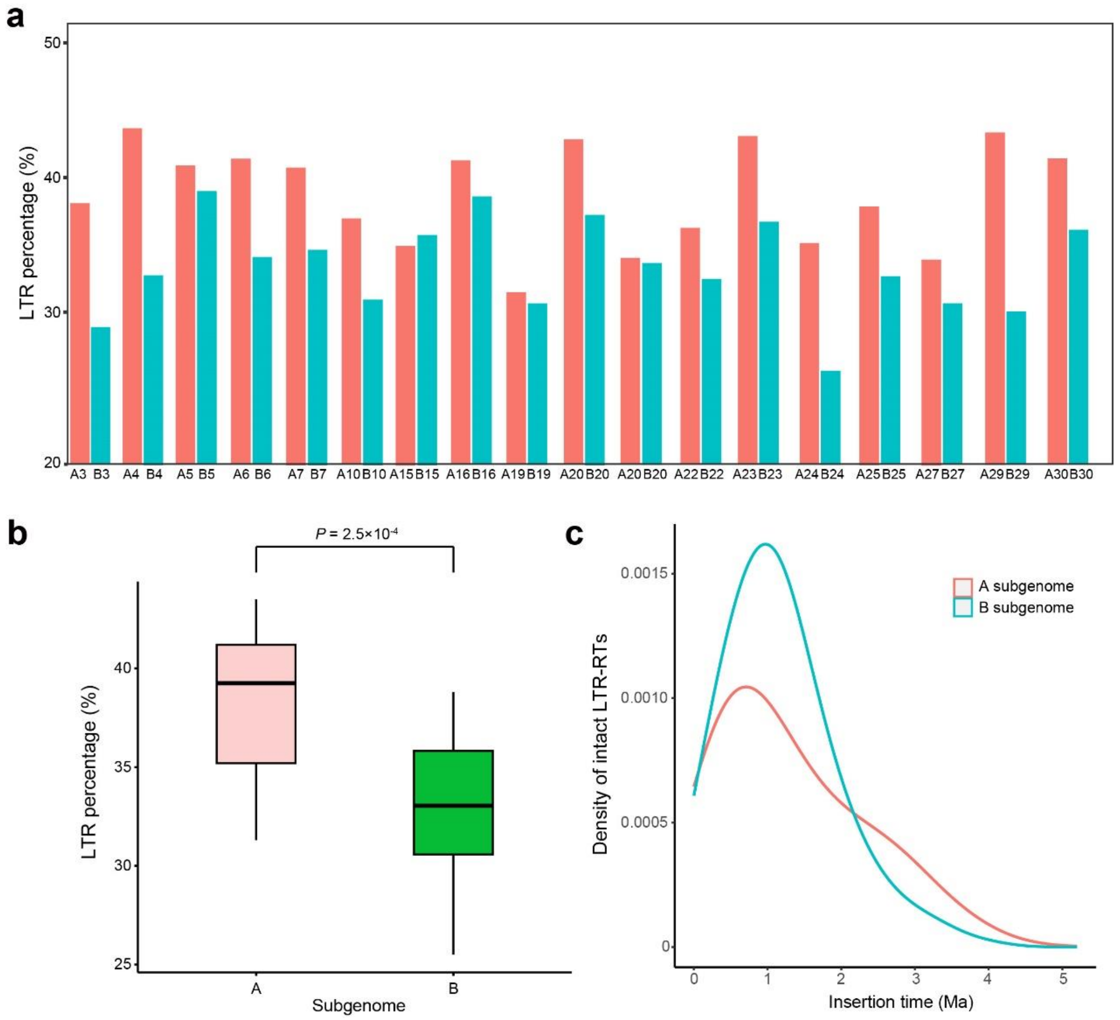

Supplementary Figure 6 Comparison of chromosomes with 1:1 syntenic relationship in the A and B subgenomes based on LTR features. (a) Histogram of LTR percentage of the chromosomes with 1:1 syntenic relationship in A and B subgenome. **(b)** LTR percentage distribution in A and B subgenome of 1:1 syntenic chromosomes. Statistical significance was determined using Wilcoxon rank sum test. **(c)** LTR insertion time of the A and B subgenomes.

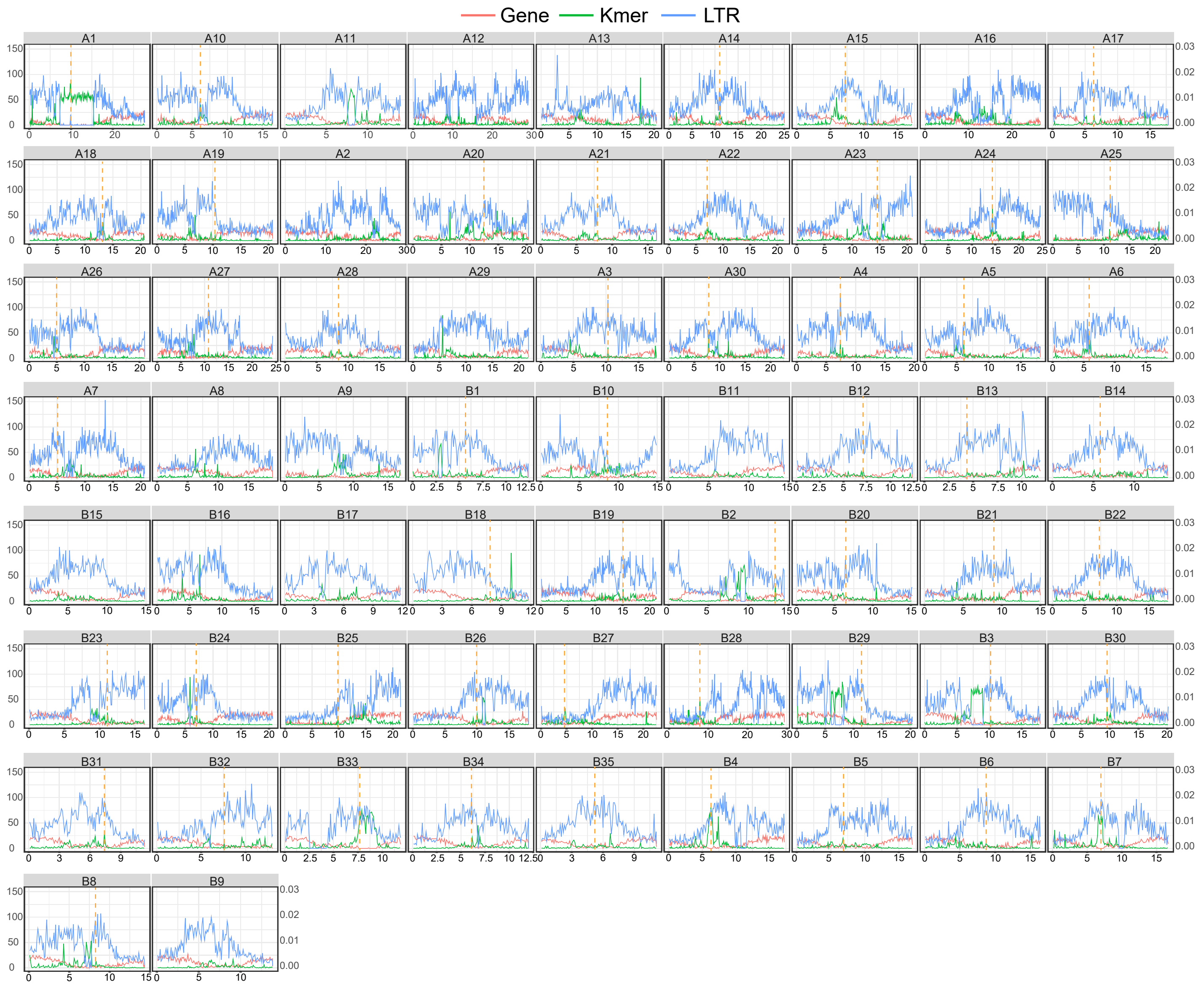

Supplementary Figure 7 Identification of the candidate centromere regions. The red, green, and blue lines represent gene density, 20-mer frequency, and LTR density, respectively.

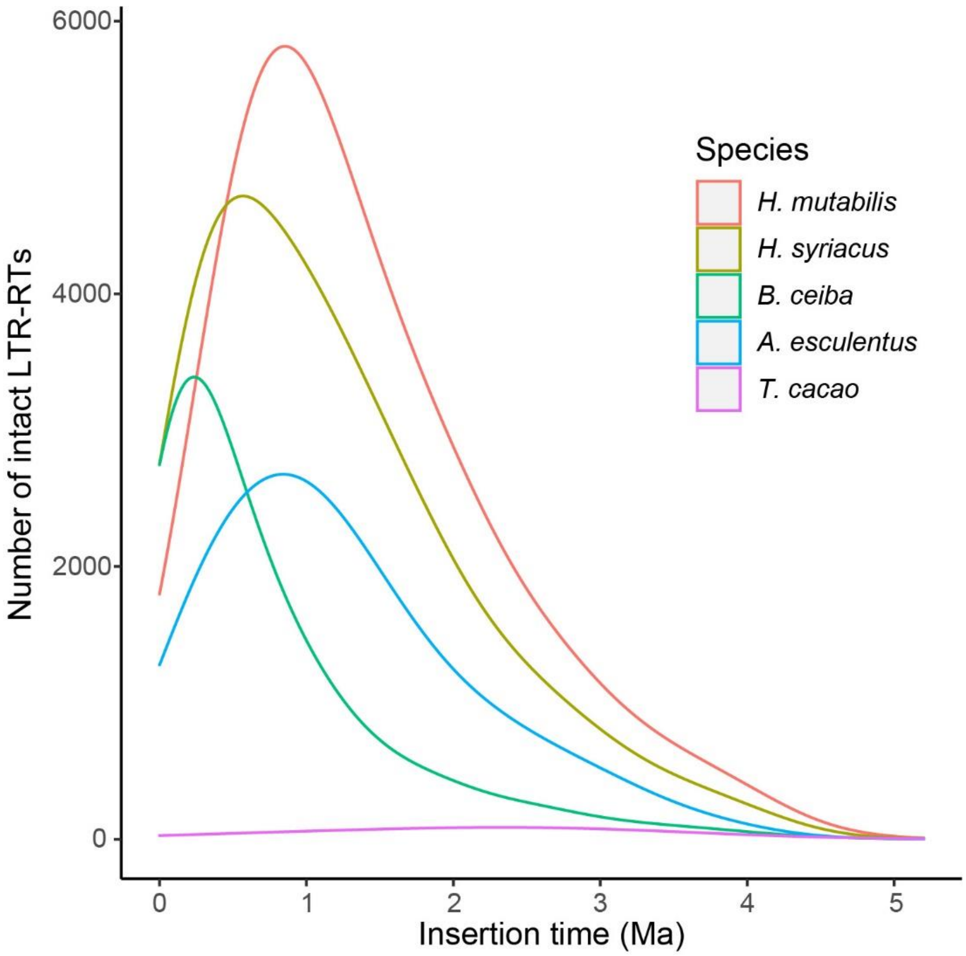

Supplementary Figure 8 Distribution of insertion times of LTR-RTs in *A. esculentus* and four closely related species.

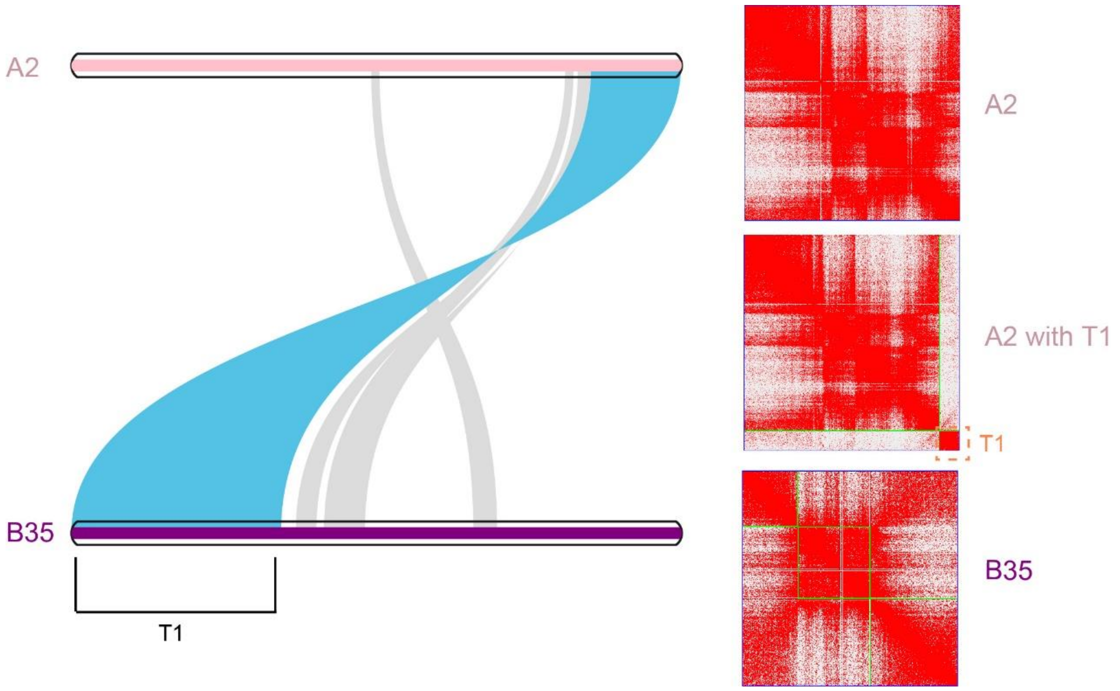

Supplementary Figure 9 The verification of translocation between A2 and B35 chromosomes of *A. esculentus* based on Hi-C interaction data.

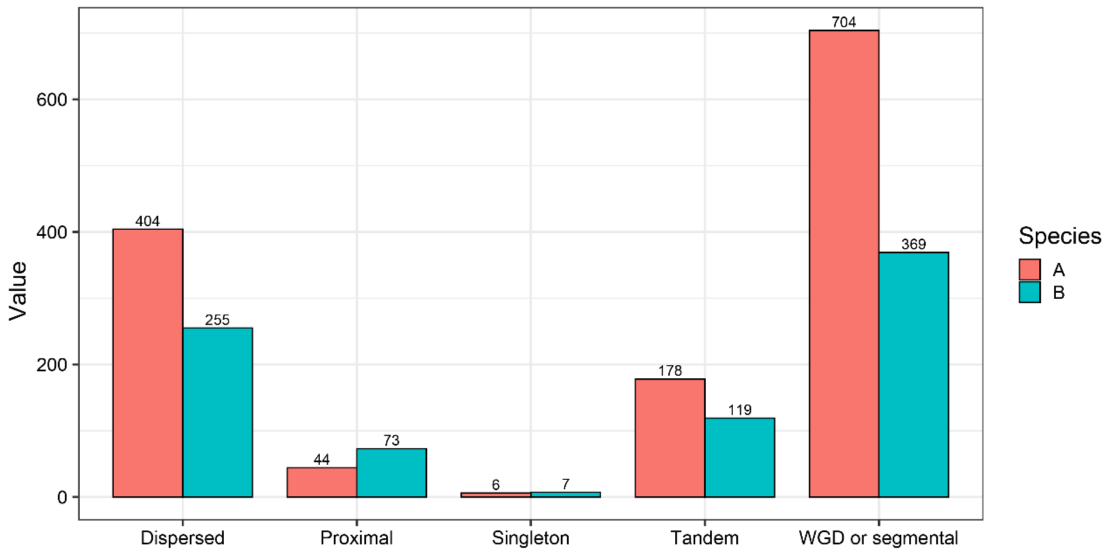

Supplementary Figure 10 The distribution of duplicate gene types for expanded gene families in the A and B subgenomes. The major duplicate type of expanded gene families in both subgenomes was WGD/segmental.

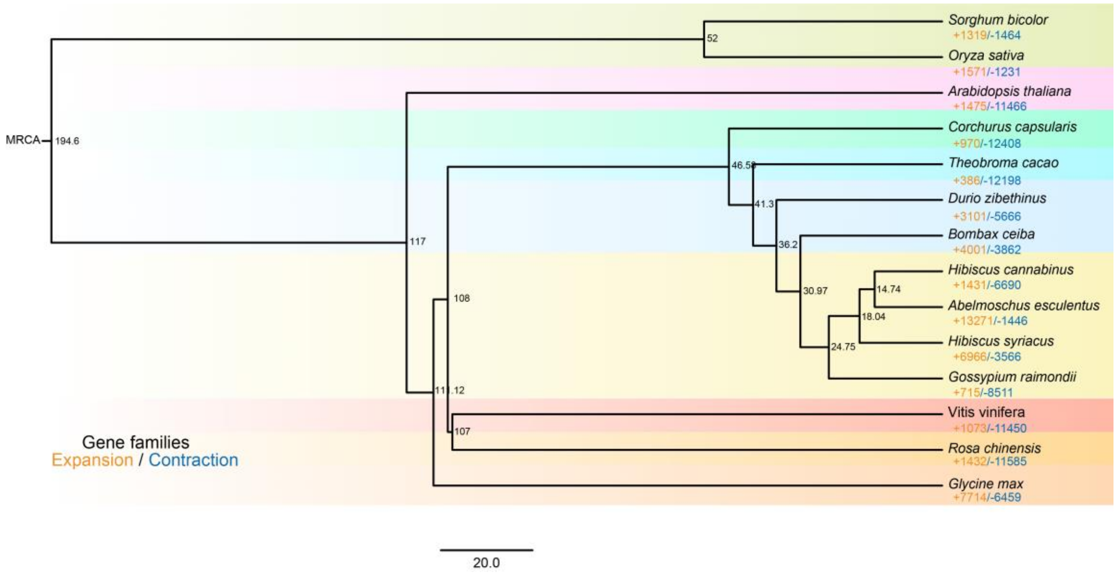

Supplementary Figure 11 Phylogenetic tree with *A. esculentus* as an individual species.

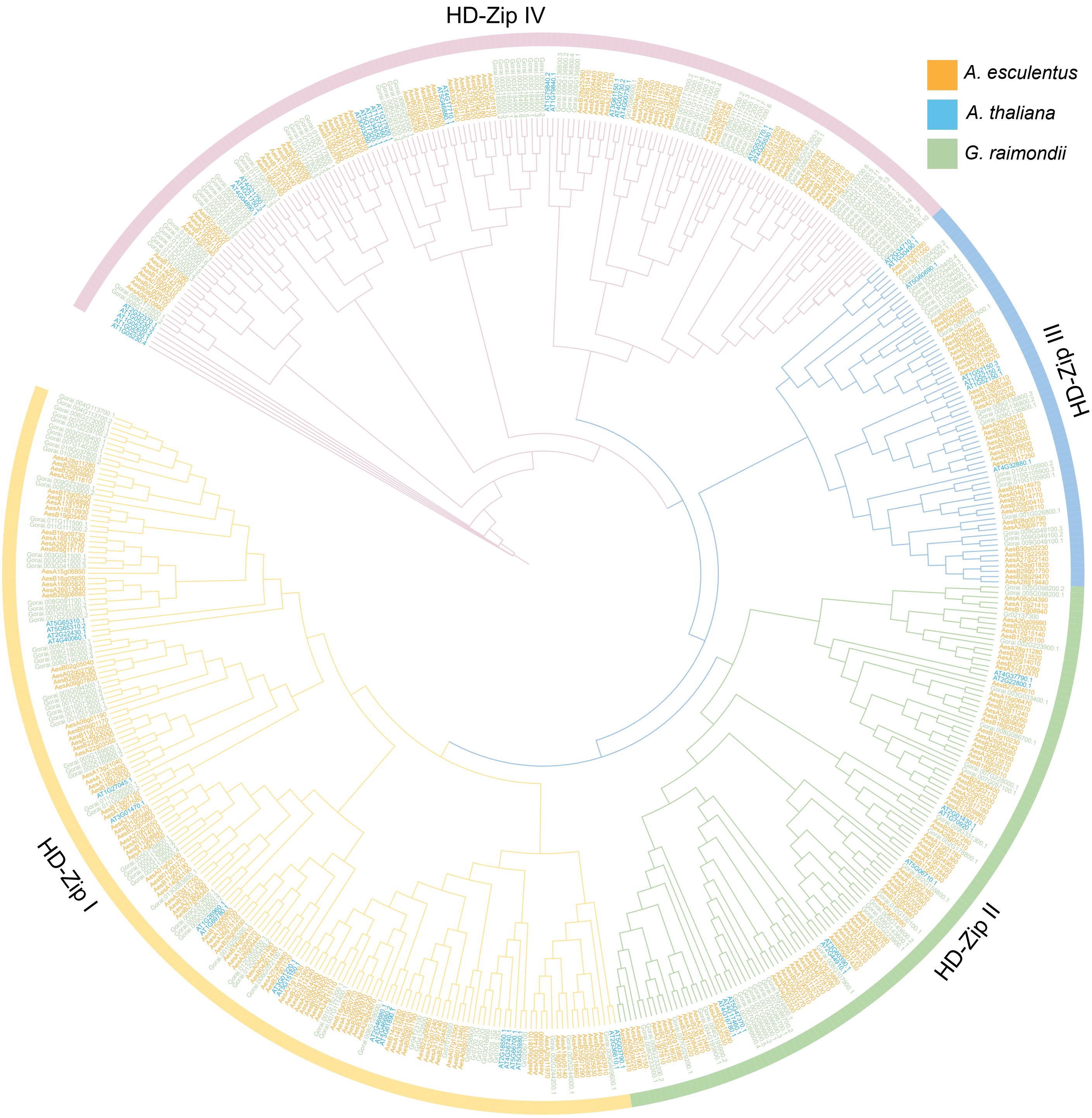

Supplementary Figure 12 Phylogenetic tree of HD-Zip transcription factors in *A. thaliana*, *G. raimondii*, and *A. esculentus* genome.

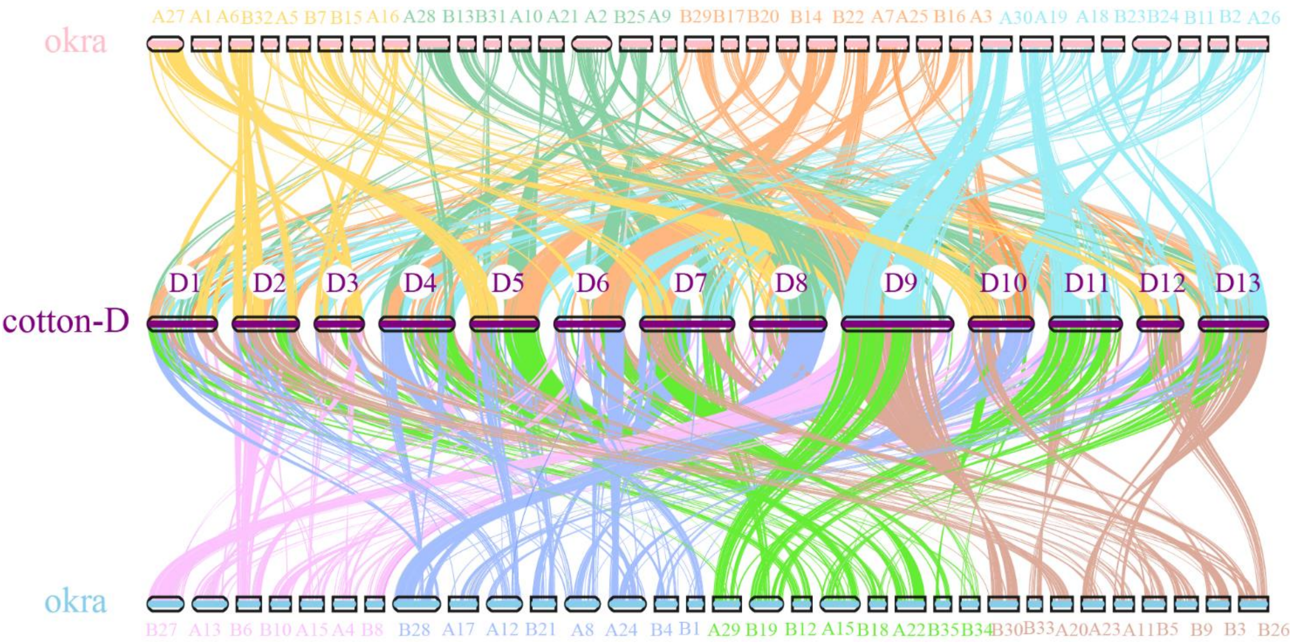

Supplementary Figure 13 Syntenic comparison between *G. raimondii* and *A. esculentus*. The 65 chromosomes of *A. esculentus* were divided into eight sets based on the syntenic results. Eight or sixteen *A. esculentus* chromosomes aligned well with one chromosome of *G. raimondii* were placed into eight groups.

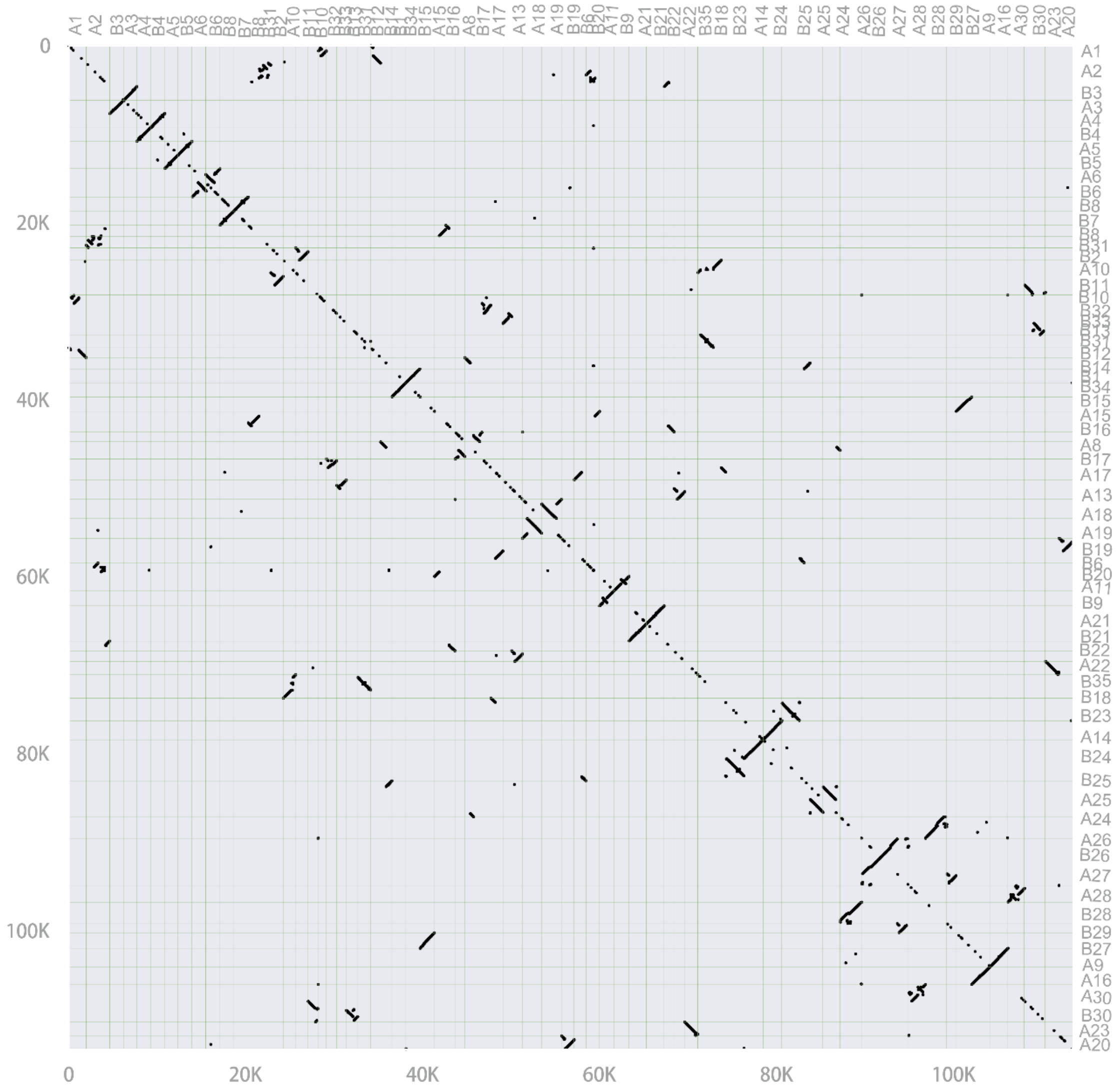

Supplementary Figure 14 Intra-genomic comparison of *A. esculentus*.

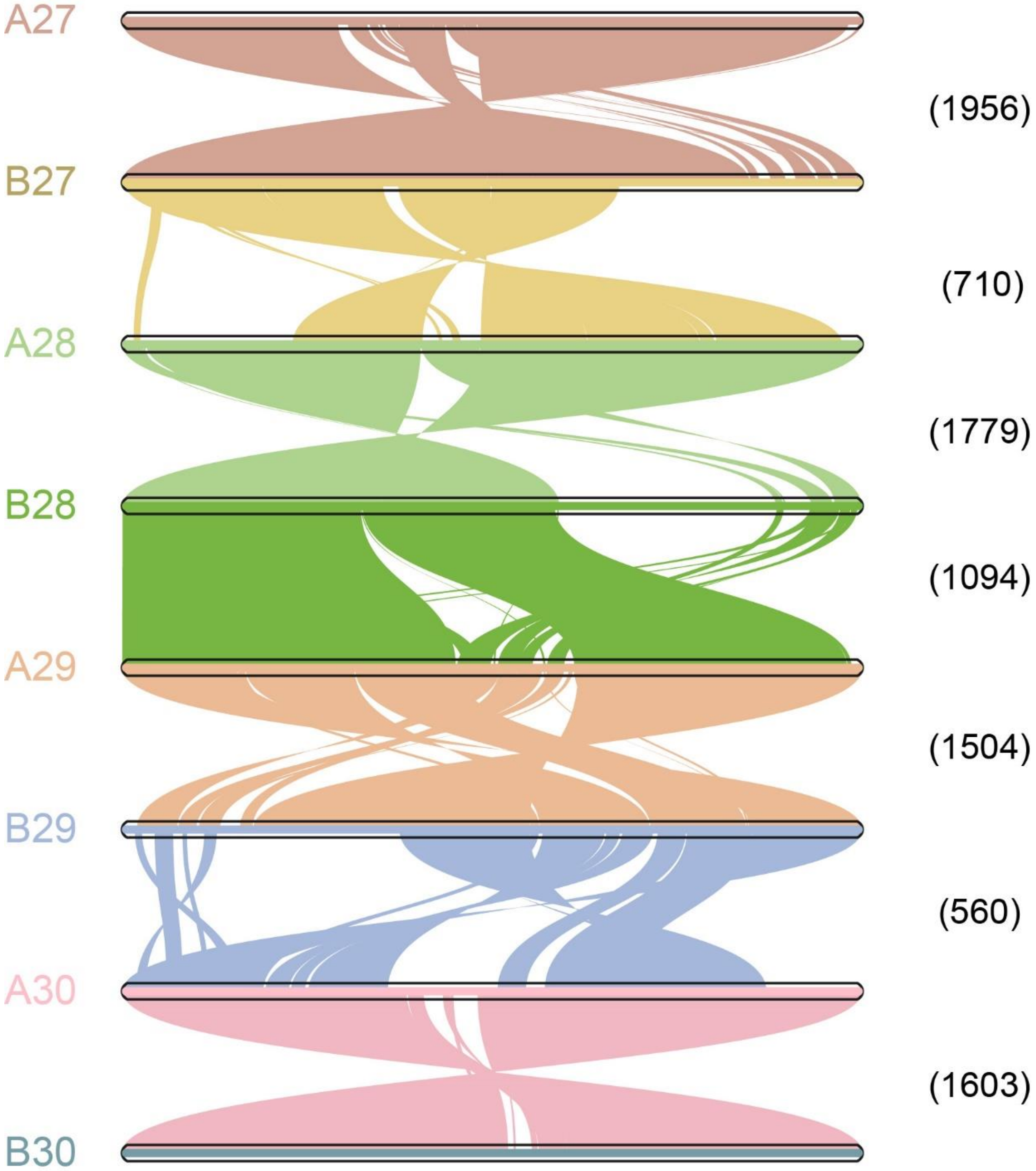

Supplementary Figure 15 Syntenic comparison between the eight *A. esculentus* chromosomes which completely aligned with *G. raimondii* D9. The numbers in parentheses represented the counts of syntenic genes between the paired chromosomes.

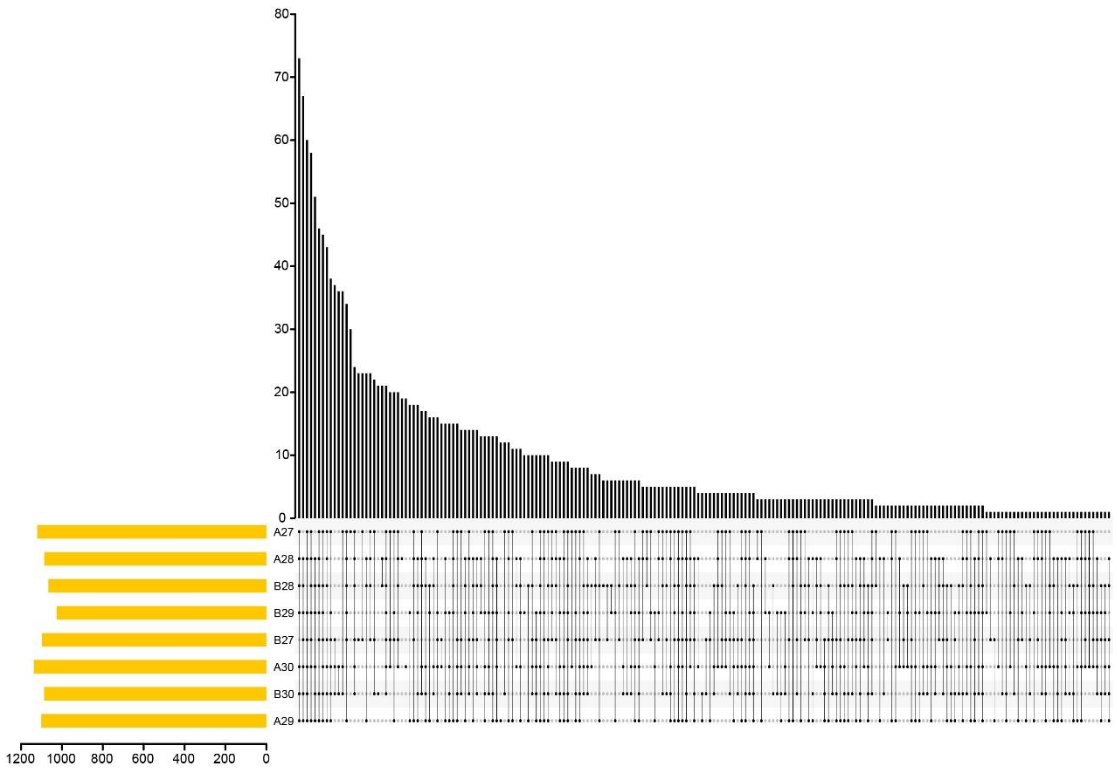

Supplementary Figure 16 The alignment results of eight chromosomes of *A. esculentus* (A27, B27, A28, B28, A29, B29, A30, and B30) against chromosome D9 of *G. raimondii* by BLAT.

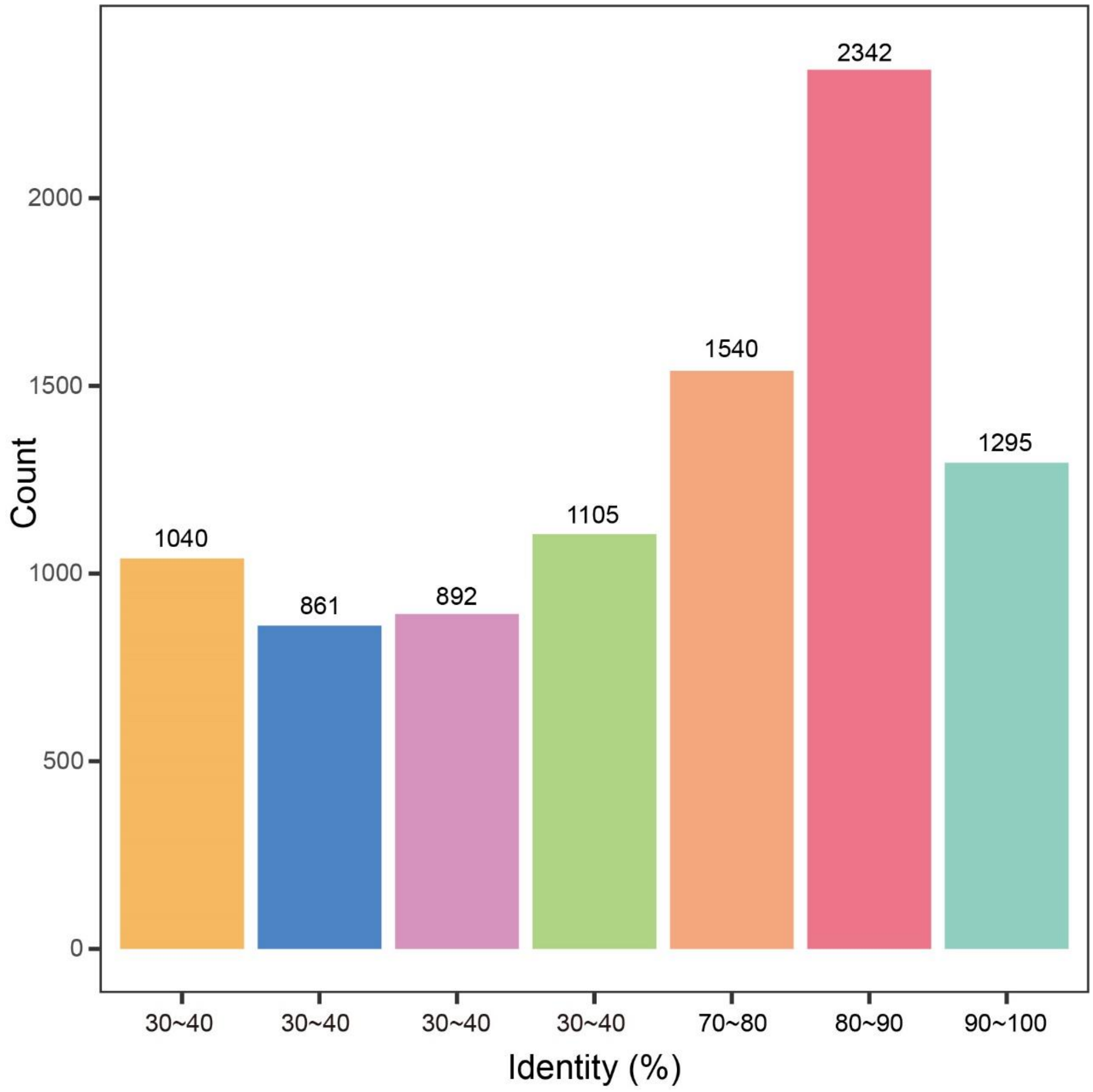

Supplementary Figure 17 The distribution of identities between the non-syntenic genes between *G. raimondii* and *A. esculentus* using BLAT.

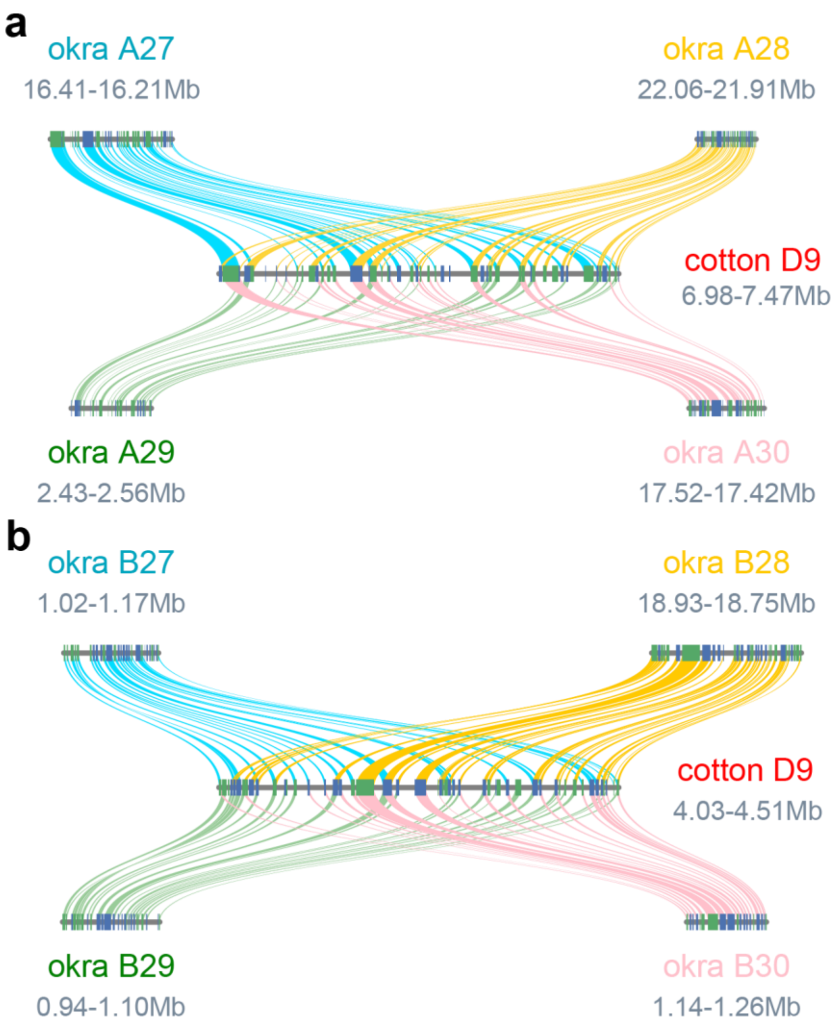

Supplementary Figure 18 Microsynteny plot showing syntenic comparison between two subgenomes and cotton D9 chromosome. (a) The result for the four chromosomes (A27, A28, A29, and A30) in the A subgenome. **(b)** The result for the four chromosomes (B27, B28, B29, and B30) in the B subgenome.

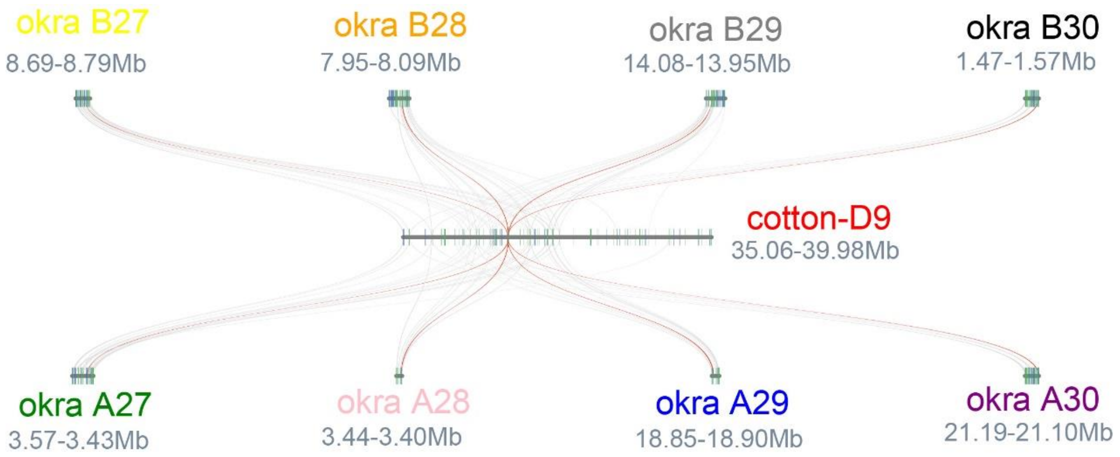

Supplementary Figure 19 An example of 1:8 relationship between *G. raimondii* and *A. esculentus.* The XM012585288.1 is located in *G. raimondii* D9 35.06M ~ 39.98M, and could be well aligned with eight genes located in different chromosomes of the okra genome, such as AesA27g04330, AesA28g05060, AesA29g14990 AesA30g18090, AesB27g08100, AesB28g17100, AesB29g11770, and AesB30g17570.

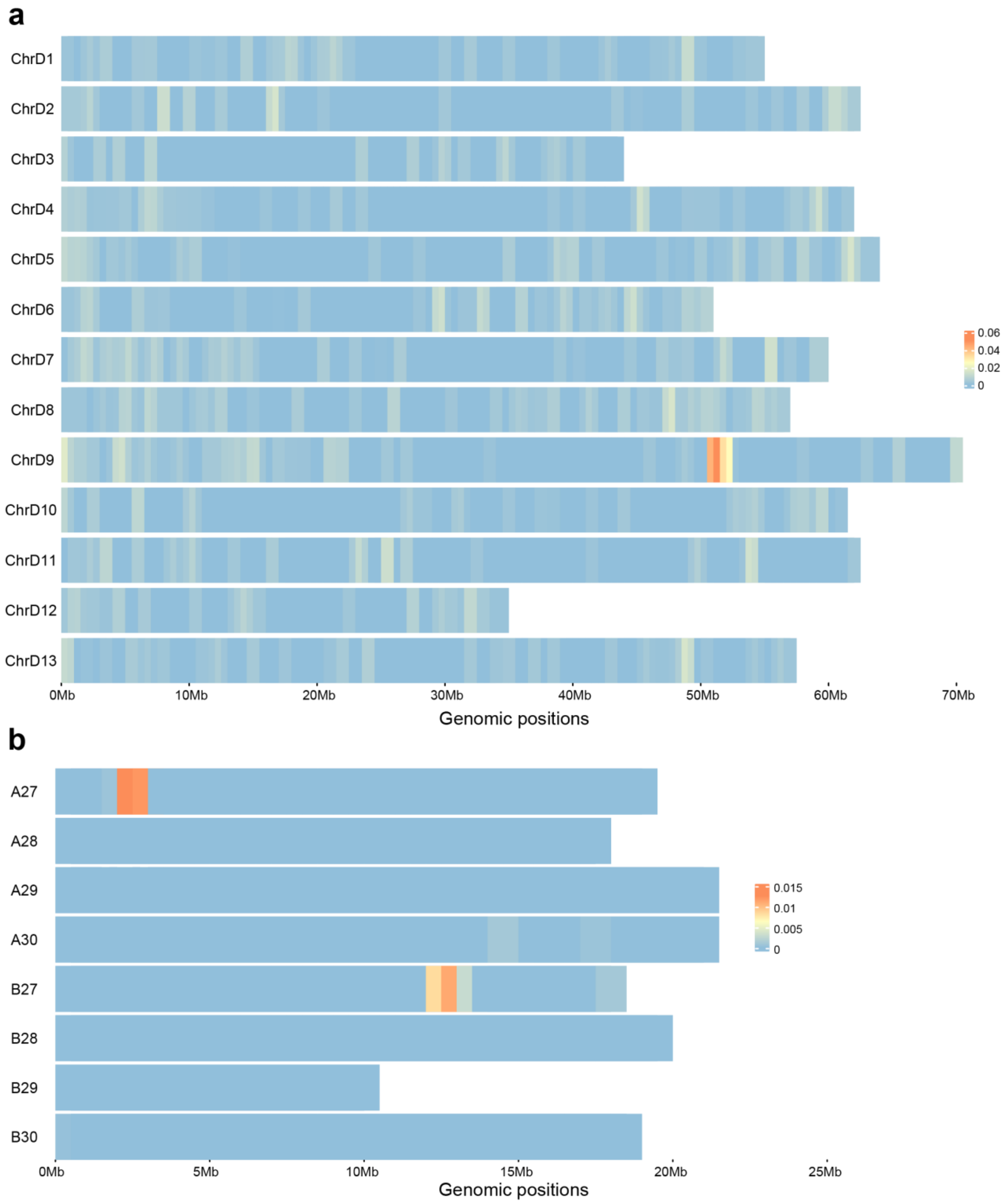

Supplementary Figure 20 Chromosomal distribution of PPR genes in *G. raimondii* and *A. esculentus*. **(a)** Distribution of PPRs in the *G. raimondii* genome. **(b)** Distribution of PPRs in the *A. esculentus* genome.

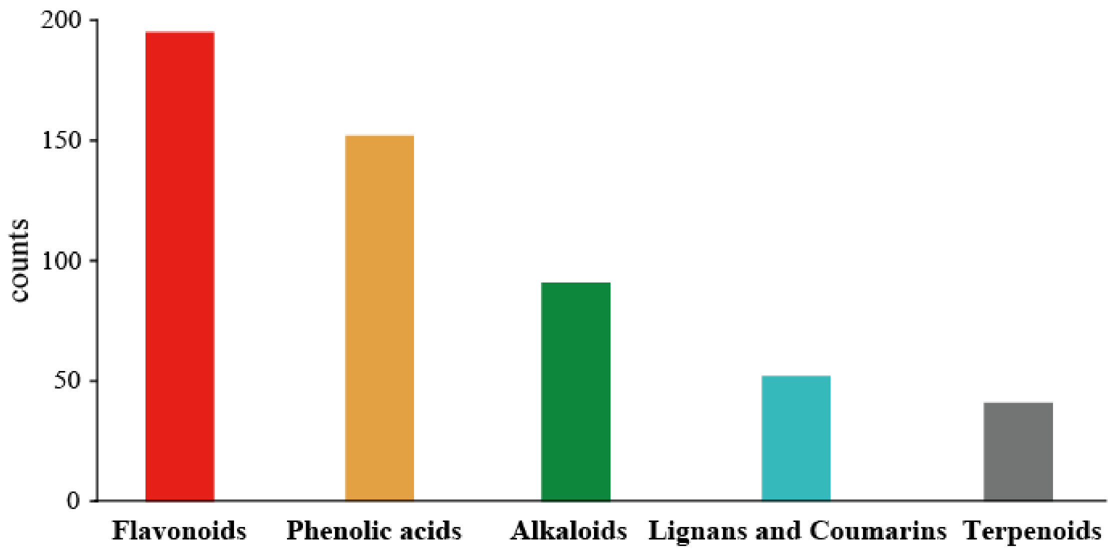

Supplementary Figure 21 Barplot showing the distribution of five major types of metabolic compounds in *A. esculentus*.

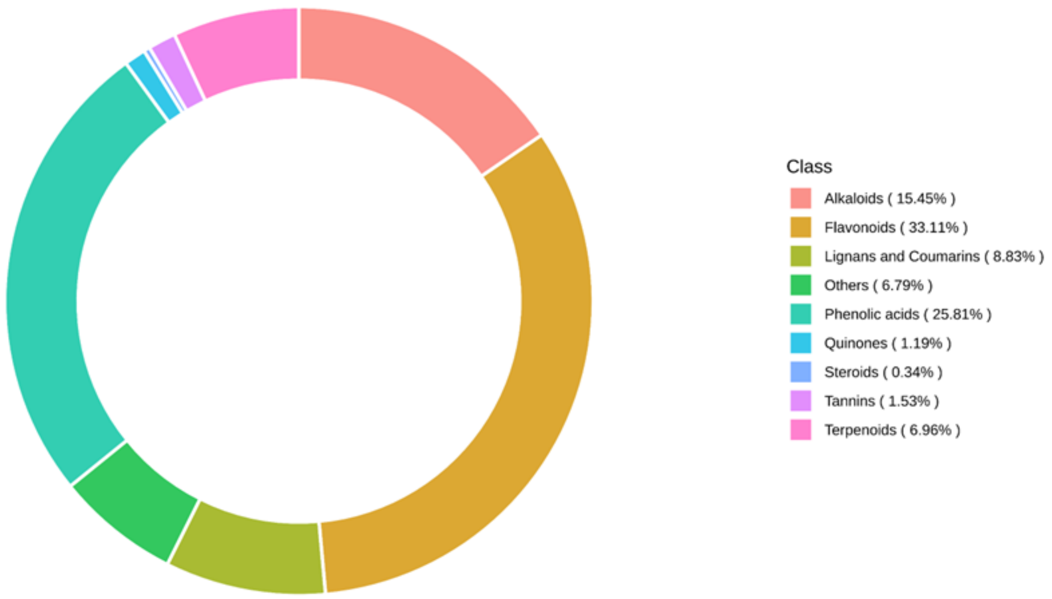

Supplementary Figure 22 Distribution of different classes of metabolites identified in *A. esculentus*.

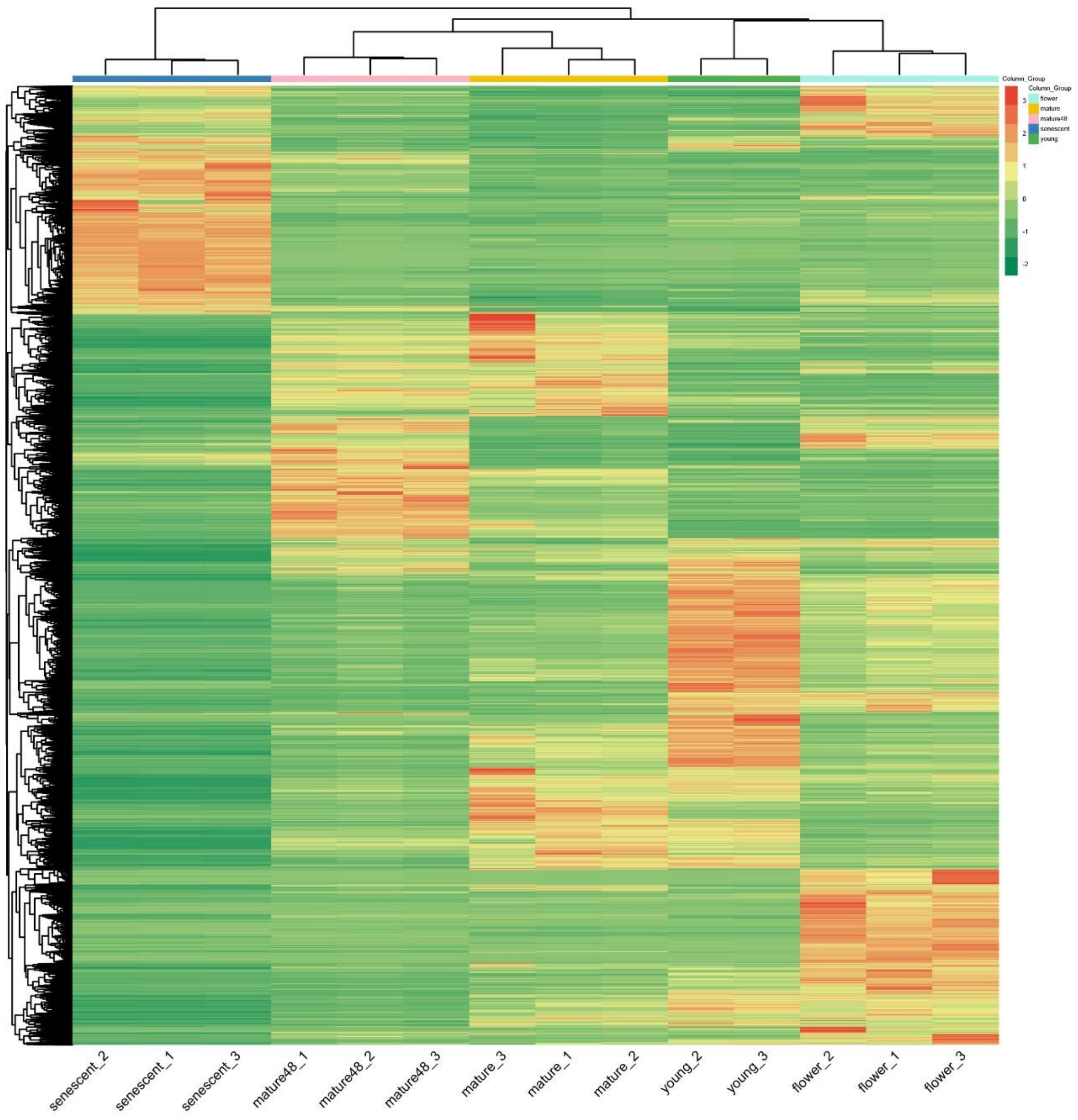

Supplementary Figure 23 Hierarchical clustering heatmap of differentially expressed genes in different tissues/stages of okra.

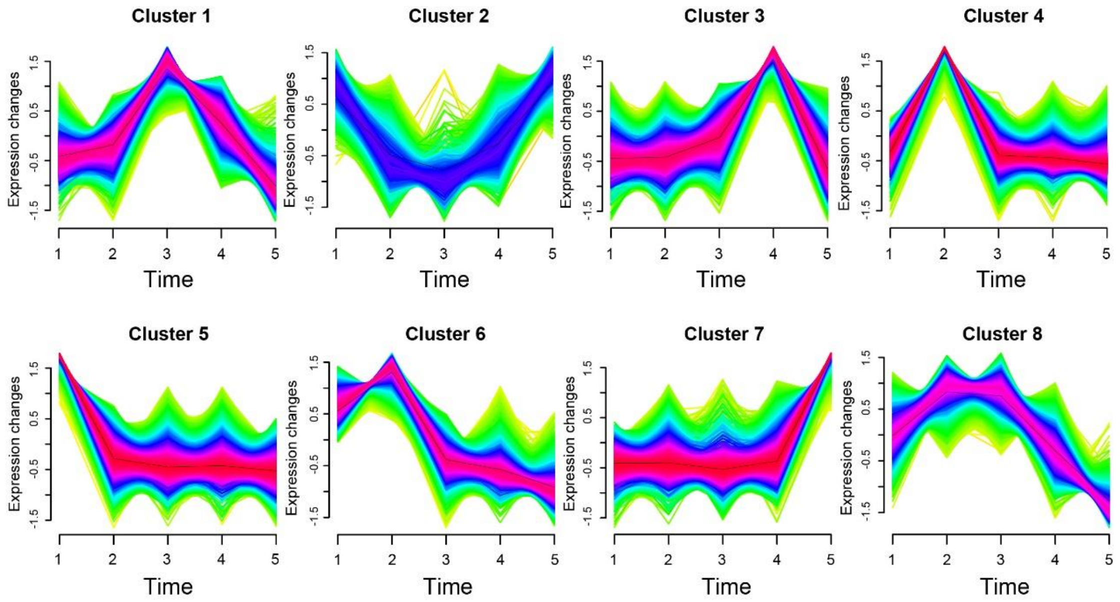

Supplementary Figure 24 Classification of differentially expressed genes based on the expression pattern through Mfuzz. The five time points in the x-axis correspond to flower, young, mature, mature-48h, and senescent pods, respectively.

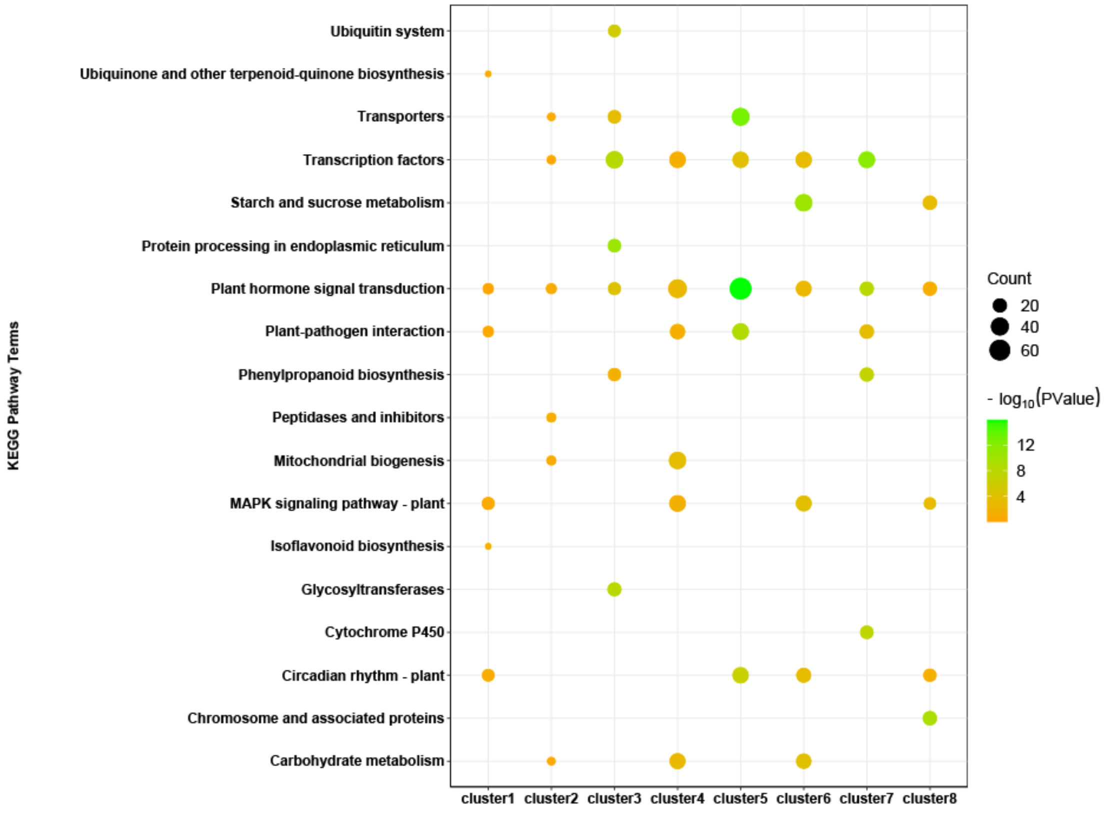

Supplementary Figure 25 KEGG enrichment result for genes in different clusters.

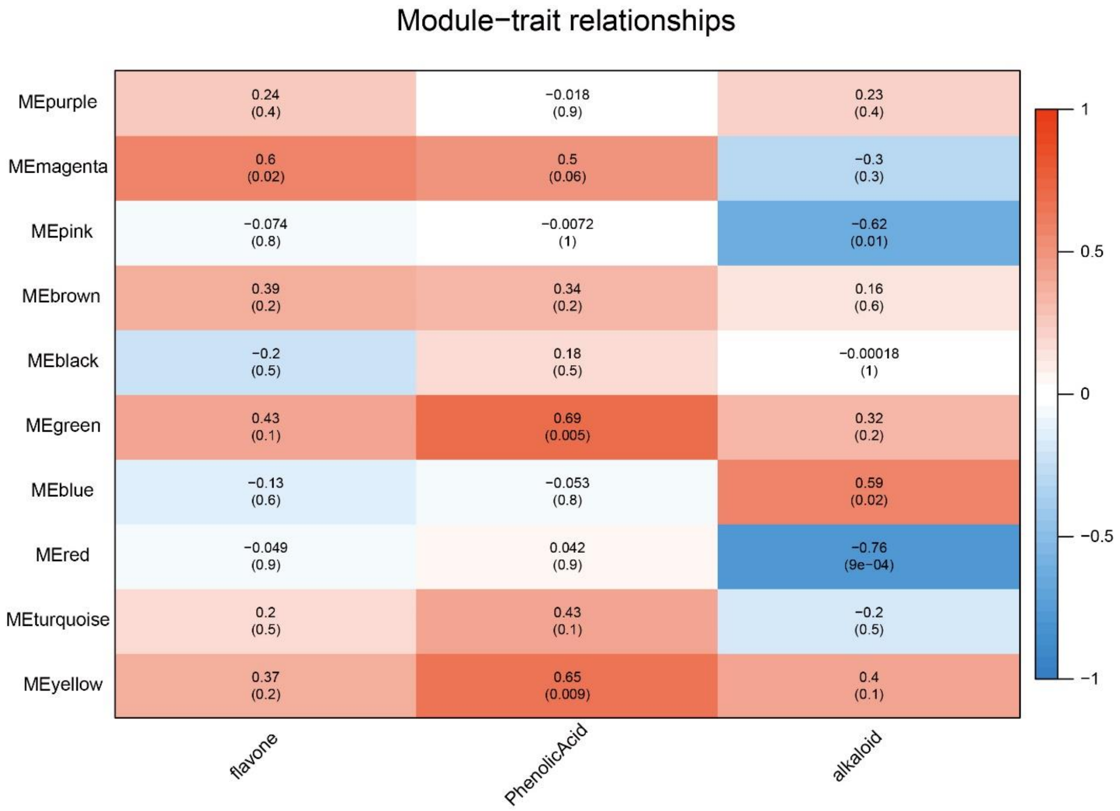

Supplementary Figure 26 Heatmap showing the relationship between each module and three types of metabolites (flavone, alkaloid, and phenolic acid).

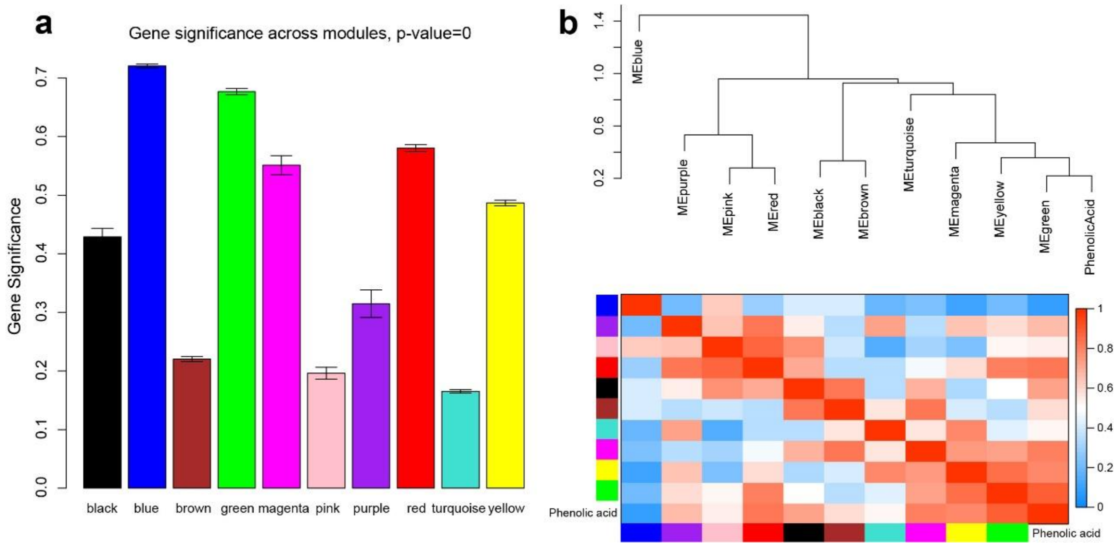

Supplementary Figure 27 WGCNA analysis of phenolic acid. The MEgreen module showed significant relationship with phenolic acid.

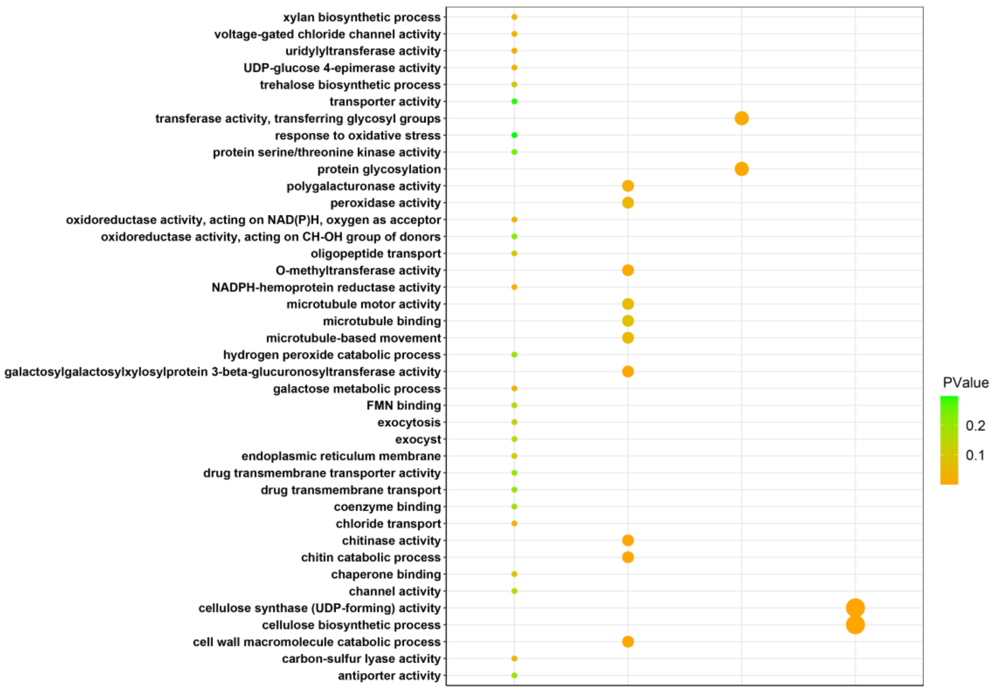

Supplementary Figure 28 GO enrichment analysis of the peripheral genes.

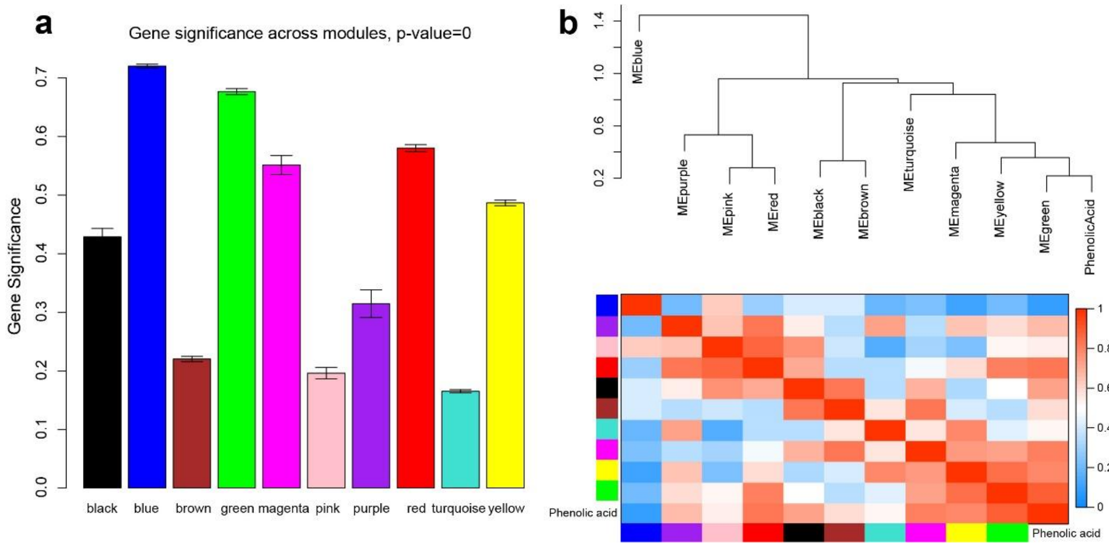

Supplementary Figure 29 WGCNA analysis of sinapyl alcohol. The MEblue module showed significant relationship with sinapyl alcohol. **(a)** Different modules with different gene significance values. **(b)** Phylogenetic tree showed that MEblue module has strong correlation with sinapyl alcohol.

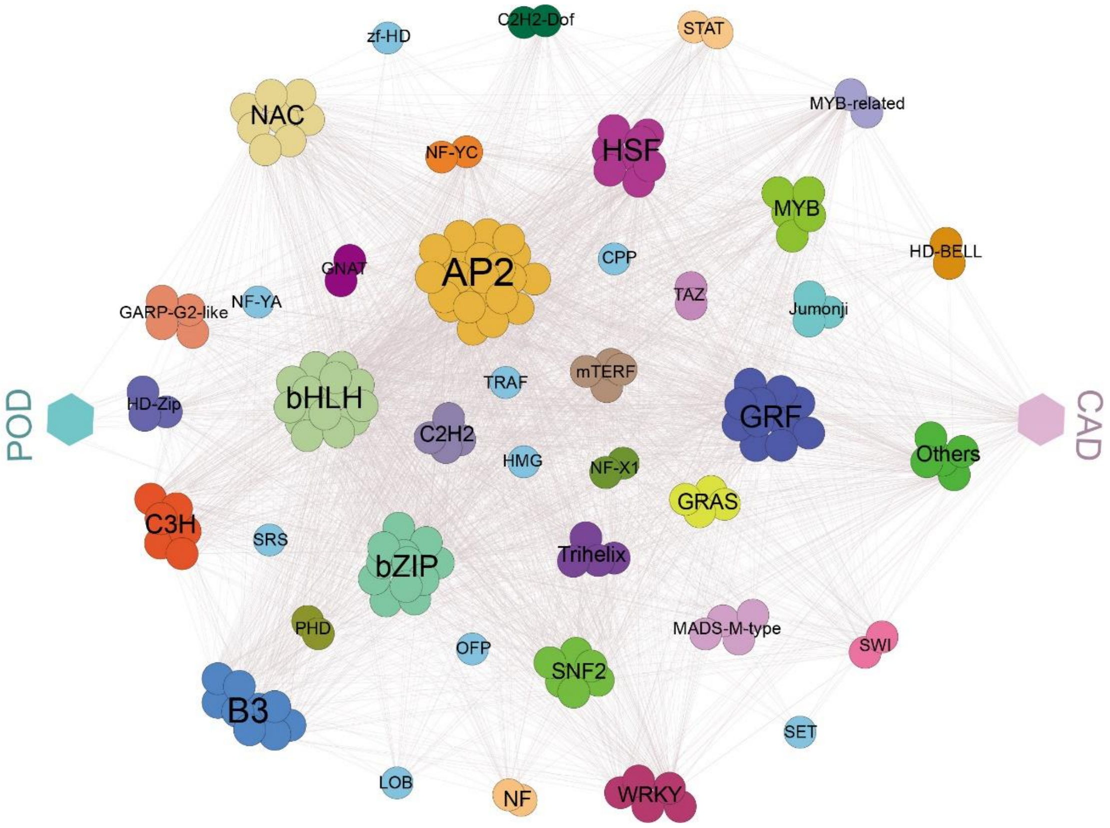

Supplementary Figure 30 Co-expression network of sinapyl alcohol with transcription factors.
